# HAX-1 interferes in assembly of NLRP3-ASC to block microglial pyroptosis in cerebral I/R injury

**DOI:** 10.1038/s41420-024-02005-3

**Published:** 2024-05-29

**Authors:** Xin-bin Guo, Xin Deng, Jingjing Wang, Yuruo Qi, Wen Zhao, Sheng Guan

**Affiliations:** 1 Department of Neuro-interventional Radiology, The First Affiliated Hospital of Zhengzhou University, 1 Jianshe Road, 450052 Zhengzhou, China; 2 Key Laboratory of Advanced Pharmaceutical Technology, Ministry of Education of China, Co-innovation Center of Henan Province for New Drug R & D and Preclinical Safety, School of Pharmaceutical Sciences, Zhengzhou University, 100 Kexue Avenue, 450001 Zhengzhou, Henan China

**Keywords:** Trauma, Hypoxic-ischaemic encephalopathy

## Abstract

Acute cerebral ischemia has a high rate of disability and death. Although timely recanalization therapy may rescue the ischemic brain tissue, cerebral ischemia-reperfusion injury has been shown to limit the therapeutic effects of vascular recanalization. Protein HAX-1 has been reported as a pro-survival protein that plays an important role in various disorders, particularly in association with the nervous system. However, the effects and mechanisms of HAX-1 in cerebral IR injury have yet to be elucidated. So, we aimed to investigate the effect of HAX-1 on microglial pyroptosis and explore its potential neuroprotective effects in ischemia-reperfusion injury. Our results show that the expression of HAX-1 decreased after cerebral IR injury, accompanied by an increase in pyroptosis pathway activation. In addition, HAX-1 could inhibit microglial pyroptosis both in vivo and in vitro and reduce the release of inflammatory mediators. The above neuroprotective effects might be partially mediated by inhibiting of interaction of NLRP3 and ASC through competitive binding, followed by the attenuation of NLRP3 inflammasome formation. In conclusion, Our findings support that HAX-1 exhibits a protective role in cerebral I/R injury, and further study on HAX-1 expression regulation will contribute to cerebral infarction therapy.

## Introduction

Acute cerebral infarction is the most common clinical cerebrovascular disease and is associated with high morbidity and mortality rates [1]. Rapid revascularization therapy is considered an effective treatment for cerebral ischemic injury. However, cerebral ischemia/reperfusion (I/R) injury often occurs inevitably in ischemic brain tissue after revascularization therapy and significantly limits neurological recovery after ischemic stroke [2–4]. Exploring the underlying mechanisms of cerebral ischemia-reperfusion injury may provide innovative treatments for acute cerebral infarction.

Cerebral I/R injury refers to the pathophysiological process of further injury and neuronal death in the ischemic areas due to reactive oxygen species (ROS) and calcium overload after the restored blood supply to the ischemic tissue [5–7]. Studies have shown that the mitochondrial pathway, apoptosis induced by the death receptor pathway, and apoptosis induced by autophagy and pyroptosis play an important role in cerebral I/R injury [8]. Recent studies have shown that pyroptosis occurs in cerebral I/R injury and is closely related to cerebral I/R injury and the inflammatory reactions in the reperfused areas [9–11]. As a pattern of programmed cell death, pyroptosis is characterized by the activation of caspase1 (the classical pathway) or caspase11 (the non-classical pathway), thus leading to the activation of gasdermin D, the formation of cell membrane pores, and the release of inflammatory factors such as IL-1 and IL-18. The accumulated effects eventually lead to neuronal death and additional inflammatory damage to the surrounding tissue [12, 13].

Microglia represents the main immune effector of the central nervous system and responds to injuries in the central nervous system. Studies have shown that there is a close association between microglial pyroptosis and inflammatory responses in the nervous system [13, 14]. As the prior study indicated, the activation of NLRP3 inflammasome in the microglia of a mouse model of Parkinson’s disease led to pyroptosis, which subsequently led to an inflammatory reaction in the nervous system and neuronal apoptosis. The authors also showed that by inhibiting the activation of NLRP3 in microglia, it was possible to alleviate the neurodegenerative changes [15]. These studies suggest that microglial pyroptosis is closely related to the inflammatory responses of the brain tissue after ischemia-reperfusion. Therefore, identifying a critical target to regulate microglial pyroptosis will likely represent a key direction and innovation in the treatment of cerebral infarction.

Hematopoietic cell-specific protein-associated protein X1 (HAX-1) protein, as an anti-apoptotic protein, was first discovered in congenital severe neutropenia. Mutation of the *HAX-1* gene leads to the loss of protein function and the premature apoptosis of neutrophils and nervous cells, thus resulting in neutropenia and neurological dysfunction [16]. In recent years, it has been found that the HAX-1 protein played an anti-apoptotic role in many biological systems [17–19]. For example, HAX-1 is involved in the regulation of neuronal synapse formation and ion channel function and plays a key role in neurodegenerative diseases [20]. In the cardiovascular system, HAX-1 protein inhibits the apoptosis of the mitochondrial pathway in cardiomyocytes and reduces the degree of myocardial infarction in mouse models by regulating mitochondrial membrane permeability (MPTP) [21]. Similarly, the latest research shows that under an ischemic and hypoxic situation, NOX2 protein in neuronal cells induces the increase of ROS, reduces the expression of HAX-1, and leads to neuronal apoptosis after cerebral ischemia [22]. In our previous studies, we also found that in endothelial progenitor cells (EPCs), HAX-1 can promote cellular tolerance to oxidative stress and thus reduce apoptosis in the mitochondrial pathway. The mechanisms underlying the protective effects of HAX-1 protein involve the combination of Hsp90 protein and AKT1 protein, thus playing an anti-apoptotic role [23]. Studies have shown that Hsp90 protein can bind to NLRP3 protein and participate in the formation of inflammasome [24, 25]. And some studies have found that HAX-1 participates in IL-1 transportation and is involved in the formation of NLRP3 inflammasome [26–29]. Therefore, we hypothesized that HAX-1 could affect the formation of NLRP3 inflammasome, regulate the process of microglia pyroptosis, protect neuronal cells, and eventually attenuate I/R injury.

Our current study demonstrates that the expression levels of HAX-1 were reduced in the ischemic area in a mouse model of cerebral I/R, while the overexpression of HAX-1 protein could reduce the area of cerebral infarction and the degree of microglia pyroptosis. Therefore, targeting the HAX-1 protein may potentially play a protective role in cerebral I/R injury. The in vitro study showed that HAX-1 protein was able to reduce the activation of Caspase 1, Gasdermin D, and the release of IL-1/18, following glucose oxygen deprivation/reoxygenation, suggestive of a microglia pyroptosis inhibition through HAX-1 protein. Moreover, HAX-1 was found to regulate the formation of NLRP3 inflammasome by inhibiting the combination of NLRP3 and ASC by competitive binding to NLRP3. In conclusion, HAX-1 protein plays a protective role in cerebral ischemia-reperfusion injury by regulating pyroptosis in microglia and the release of inflammatory mediators, thus suggesting that HAX-1 protein presents a novel therapeutic target for cerebral infarction.

## Results

### The extent of pyroptosis in microglia was increased in the cerebral infarction area in the mouse model of tMCAO

**Fig. 1:**
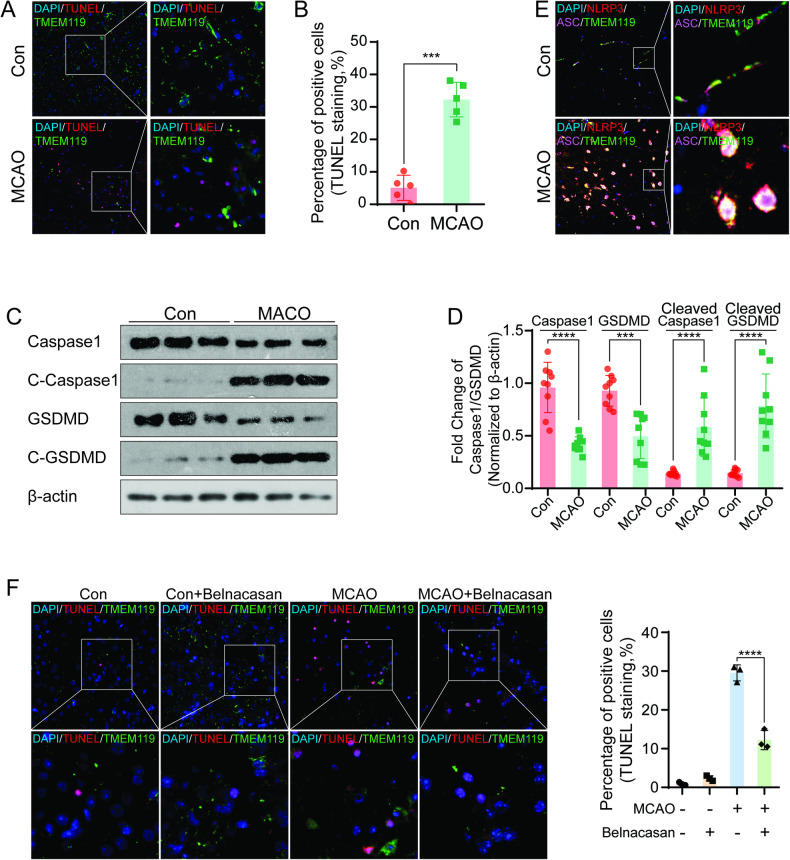
Microglial pyroptosis in the cerebral infarction area in a mouse model of tMCAO. **A**, **B,** and **F** IF and TUNEL staining. Apoptotic cell nuclei were labeled with red fluorescence, all nuclei were stained with blue fluorescence, and TMEM119-positive cells were labeled with green fluorescence (magnification: ×40 and ×100). **C** and **D** Western blotting showed that Caspase1 and Gasdermin-D were activated after MCAO modeling. β-actin was used as a loading control. **E** Confocal microscopy revealed the colocalization of NLRP3 and ASC. NLRP3 was stained with red fluorescence, ASC was stained with purple fluorescence, TMEM119 was stained with green fluorescence, and nuclei were stained with blue fluorescence (magnification: ×40 and ×600). ***p < 0.001; ****p < 0.0001.

The H&E-stained slides from the mouse brain sections from tMCAO showed the confirmed areas of infarction (S-Fig. 1A). Then, we detected nerve cell apoptosis in the infarct area and investigated its correlation with microglia by TUNEL staining and immunofluorescence staining methods. We observed obvious nerve cell apoptosis in the infarcted areas in the experimental group and the presence of TMEM119-positive cells near the apoptotic cells, thus suggesting that the apoptotic nerve cells were spatially related to the microglia (Fig. 1A and B). Then, we detected the activation of the pyroptosis pathway in cerebral infarction tissue by Western blotting. Compared with the uninjured contralateral brain tissue, the levels of pro-caspase1 and pro-Gasdermin-D in the infarct area had decreased, while the levels of cleaved aspase1 and Gasdermin-D proteins had increased significantly, thus indicating that the pyroptosis pathway had been activated in infarcted brain tissues (Fig. 1C and D). In order to further clarify the correlation between the activation of the pyroptosis pathway and the microglia in infarcted areas, the co-localization of the microglia marker TMEM119 and the components of the inflammasome (NLRP3 and ASC protein) were tested by immunofluorescence staining. The formation of the inflammasome in microglia was detected in infarcted areas, and the levels of NLRP3 and ASC proteins were significantly increased with co-localization within TMEM119-positive cells. In uninjured brain tissues, NLRP3 and ASC proteins were expressed in TMEM119 cells without enhancement or co-localization. Moreover, the activation of the inflammasome in the infarcted area was highly consistent with the distribution of TMEM119, suggestive of the central role of microglia pyroptosis in the infarcted areas (Fig. 1E and S-Fig. 1B). Furthermore, the experimental animals were administered with the Caspase1 selective inhibitor belnacasan to study the changes in nerve cells apoptosis in the infarct areas. TUNEL staining showed that there was apparent nerve cell apoptosis in the cerebral infarction area in the tMCAO group, which was not detected in the control group with or without belnacasan. However, the rate of apoptosis decreased after belnacasan administration in tMCAO (Fig. 1F). These results showed that microglial pyroptosis occurred following cerebral I/R injury in mice and promoted nerve cell apoptosis in infarcted areas.

### The expression levels of HAX-1 protein were reduced in the cerebral infarction area and could affect microglial pyroptosis

**Fig. 2:**
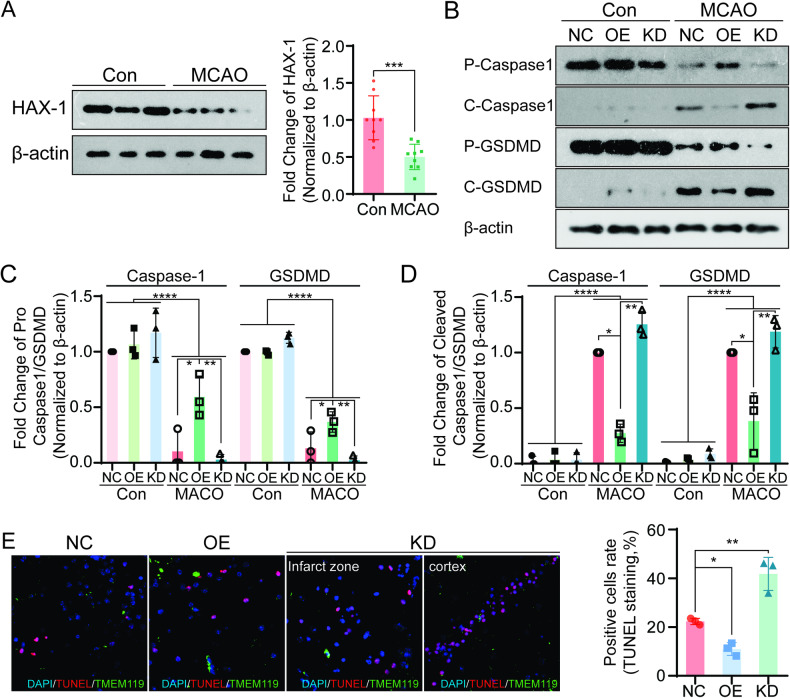
Expression levels of HAX-1 were reduced in the MCAO model and affected pyroptosis in microglia. **A** Western blotting indicated a reduction in HAX-1 expression after MCAO modeling. β-actin was used as a loading control. **B**–**D** Western blotting showed that HAX-1 regulated the activation of Caspase1 and Gasdermin-D. β-actin was used as a loading control. **E** TUNEL staining revealed different levels of apoptosis in nerve cells with different levels of HAX-1 expression. Apoptotic cell nuclei were labeled with red fluorescence, all nuclei were stained with blue fluorescence, and TMEM119-positive cells were labeled with green fluorescence (magnification: ×40). *p < 0.05; **p < 0.01, ***p < 0.001; ****p < 0.0001.

We found that microglial pyroptosis in the cerebral infarction zone of the tMCAO model was related to neuronal apoptosis. To further clarify the role of microglial pyroptosis in cerebral I/R injury, we studied the expression of HAX1 before and after cerebral infarction by western blotting, and the results showed that the expression of HAX-1 protein in the infarcted area was significantly lower than that in normal brain tissue (Fig. 2A). To further clarify the role of HAX-1 in cerebral I/R injury, we manipulated the expression of HAX-1 in the mouse brain by adeno-associated virus transfection. Western blotting was used to detect the intervention results, which showed that the HAX-1 overexpression AAV significantly increased the expression of HAX-1 in brain tissue, while the HAX-1 knockdown AAV reduced the expression of HAX-1 (S-Fig. 2A). The effects of HAX-1 expression on cerebral infarction following tMCAO were detected by TTC staining and H&E staining methods in three HAX-1 groups. Compared with the negative control, the infarcted areas of the HAX-1 overexpression group were reduced, while the infarct area had increased significantly in the HAX-1 knockdown group. In particular, the cerebral infarction zone had spread to the cortex when HAX-1 was knocked down (S-Fig. 2B, C). Next, we explored the effects of HAX-1 on the pyroptosis pathway in infarcted tissue. Compared with uninjured brain tissue, the levels of Caspase1 and Gasdermin D protein in infarcted tissue had decreased, while the levels of cleavage protein had increased by the western blotting method. Although the levels of Caspase1 and Gasdermin D decreased after cerebral infarction in all three groups, the expression levels of Caspase1 and Gasdermin D were higher in the HAX-1 overexpression group than those in the control group, and the knockout group showed the lowest levels of expression. We also found that the levels of Cleaved caspase1 and Cleaved gasdermin D expression were the highest in the infarcted tissue in HAX-1 knockdown mice and the lowest in the HAX-1 overexpression mice (Fig. 2B–D). Next, the effects of HAX-1 levels on nerve cell apoptosis were studied in the infarcted area. TUNEL staining showed that compared with the negative control group, the density of TUNEL staining was significantly reduced in the cerebral infarction of the HAX-1 overexpression group; while the highest expression was observed in the HAX-1 knockdown group. Cortical neurons also showed obvious apoptosis in the HAX-1 knockdown group; this was not easily observed in the other two groups. Combined with TMEM119 immunofluorescence staining, we found that nerve cell apoptosis was related to the distribution of microglia (Fig. 2E). These results indicated that HAX-1 protein played a protective role in cerebral infarction tissue by reducing the activation of the pyroptosis pathway, and attenuation of nerve cells apoptosis, in associated with microglial activities.

### HAX-1 regulated the microglial pyroptosis pathway and inflammasome formation

**Fig. 3:**
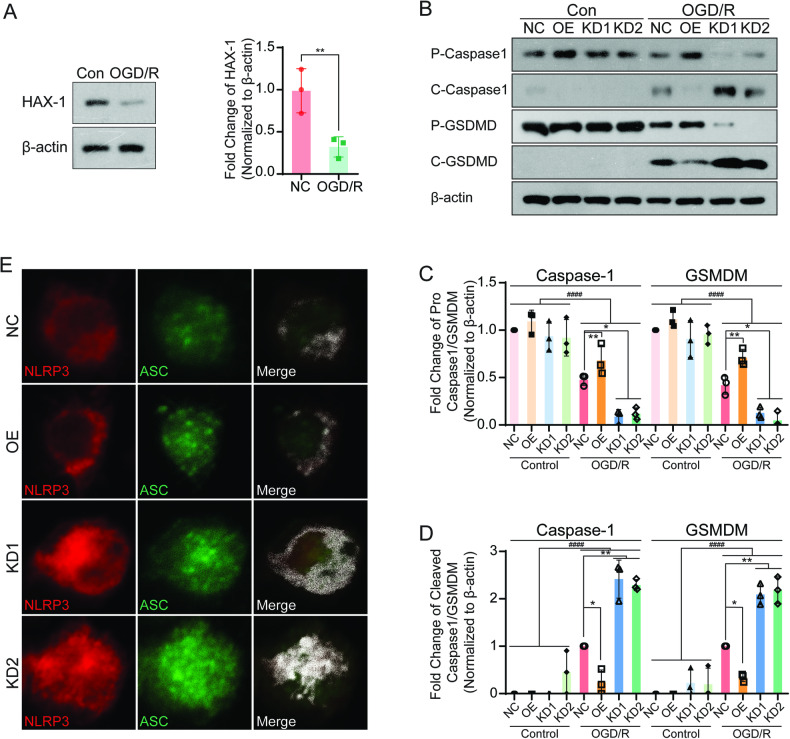
HAX-1 regulated the pyroptosis pathway and inflammasome formation in microglia. **A** Western blotting indicated that HAX-1 expression decreased after OGD/R modeling. β-actin was used as a loading control. **B**–**D** Western blotting indicated that HAX-1 regulated the activation of Caspase1 and Gasdermin-D. β-actin was used as a loading control. **E** Confocal microscopy demonstrated the colocalization of NLRP3 and ASC. NLRP3 was stained with red fluorescence, and ASC was stained with green fluorescence (magnification: ×1000). *p < 0.05; **p < 0.01, ^####^*p* < 0.0001.

In order to further clarify the correlation between the roles of HAX-1 in cerebral I/R injury and microglial functions, we used the microglia cell line BV2 culture in vitro and simulated I/R injury by glucose and oxygen deprivation/reoxygenation (OGD/R) experiments. We determined the changes in HAX-1 expression in BV2 cells before and after OGD/R modeling. We found that the expression of HAX-1 in the microglia decreased after modeling, which was consistent with the above in vivo results (Fig. 3A). Next, we manipulated the expression of HAX-1 in microglia by adenovirus transfection, which was successfully confirmed by western blotting results. The expression of HAX-1 in microglia was upregulated in the overexpression group but was decreased in the two knockdown groups (S-Fig. 3A, B). Microglia in the negative control group (NC), overexpression group (OE), and knockdown group (KD1 and KD2) were tested by OGD/R, and the activation of pyroptosis-related pathways was detected. The results were similar to those obtained from the in vivo experiments in which the expression of Caspase1 and Gasdermin D decreased after OGD/R. Compared with the NC group, the reduction of caspase1 and Gasdermin D was more prominent in the KD1 and KD2 microglia, while the declining trend was inhibited in the OE group. The expression of Cleaved Caspase1 and Gasdermin-D showed the opposite trend with prolonging. Compared with the NC group, the increased expression levels of cleaved caspase1 and Gasdermin-D were inhibited in microglia of the OE group; while the cleavage bands in the KD1 and KD2 groups were significantly increased (Fig. 3B–D). Furthermore, we also detected the release of IL-1 and IL-18 from microglia by ELISA. The results were consistent with the trend of activation in the pyroptosis pathway. Before modeling, the levels of IL-1 and IL-18 in the supernatant of each group of microglia were at a low level. After OGD/R, the levels of IL-1 and IL-18 in the supernatant of each group of microglia had increased. Compared with the NC group, the levels of IL-1 and IL-18 were higher in HAX-1 knockdown cells; while decreasing the HAX-1 overexpression manipulations (S-Fig. 3C and D). Finally, we identified the effects of HAX-1 on the formation of the inflammasome in microglia after OGD/R by immunofluorescence study. After OGD/R modeling, the aggregation of NLRP3 and ASC protein was noted in the cells of each group, although the degree of accumulation was different. Compared with the NC group, there was a slight more aggregation of NLRP3 and ASC in the OE group, although NLRP3 and ASC showed strong polymerization in the whole cell range of KD1 and KD2 microglia (Fig. 3E and S-Fig. 3E). These results suggested that HAX-1 can inhibit the formation of the inflammasome and the activation of the pyroptosis pathway in microglia and reduce the release of both IL 1 and IL 18.

### The inhibition of NLRP3 blocked the effects of HAX-1 on the pyroptosis pathway in microglia and HAX-1 regulated the formation of the inflammasome and the activation of the pyroptosis pathway in microglia by regulating the binding of ASC to NLRP3

**Fig. 4:**
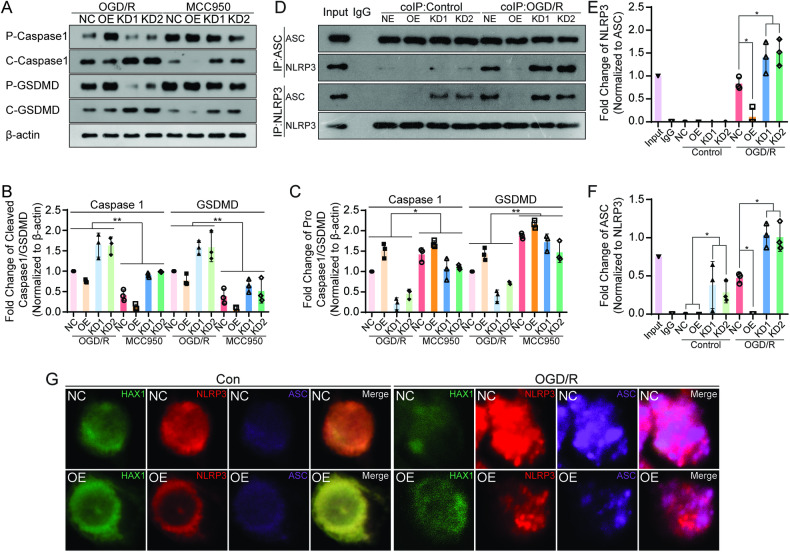
HAX-1 regulated inflammasome formation in microglia via NLRP3 inflammasome. **A**–**C** Western blot showed the inhibition of NLRP3 blocked the effects of HAX-1 on the activation of Caspase1/Gasdermin D in microglia, β-actin was used as a loading control. **D**–**F** Co-immunoprecipitation (Co-IP) showed that HAX-1 affected the interaction between NLRP3 and ASC. **G** Confocal microscopy demonstrated the colocalization of HAX-1, NLRP3, and ASC. HAX-1 was stained with green fluorescence, NLRP3 was stained with red fluorescence, and ASC was stained with purple fluorescence (magnification: ×1000). *p < 0.05; **p < 0.01, ***p < 0.001.

To further investigate the effects of HAX-1 on pyroptosis, we used a selective NLRP3 inhibitor, MCC950(APExBIO), in the microglia to investigate the effects of HAX-1 on the pyroptosis pathway. As shown in Fig. 4A, B, the levels of pro-Caspase1 and Gasdermin-D were significantly increased after the inhibition of NLRP3 in the OGD/R model. In the HAX-1 KD1 and KD2 groups, the levels of pro-Caspase1 and Gasdermin-D increased by 5.05-, 2.44-, 4.35-, and 2.03-folds, respectively, after the inhibition of NLRP3. Correspondingly, the expression levels of cleaved Caspase1 and cleaved Gasdermin-D were decreased. Comparisons among the different groups featuring NLRP3 inhibition showed that the expression levels were diminished for both pro-Caspase1/Gasdermin D and cleaved Caspase1/Gasdermin D (Fig. 4A, B). These results illustrated that NLRP3 played a crucial role in the regulation of the pyroptosis pathway through HAX-1.

To further clarify the mechanisms underlying the action of HAX-1 protein on microglial pyroptosis, co-immunoprecipitation (Co-IP) assays were performed to verify the presence of HAX-1 binding to NLRP3. Co-IP showed that HAX-1 and NLRP3 had co-precipitated in the control group, while this interaction was reduced in the OGD/R group (S-Fig. 4A, B). Next, we performed Co-IP and immunofluorescent colocalization assays to study whether HAX-1 affected the interaction between ASC and NLRP3. Co-IP results showed that compared with the control groups, the interaction between NLRP3 and ASC was much higher in the OGD/R groups. Among the OGD/R groups, NLRP3 immunoprecipitated by ASC was increased by 1.64- and 1.77-fold in the HAX-1 KD1 and KD2 groups and decreased by 0.13-fold in the HAX-1 OE group when compared with the NC groups, and the same trend was observed with baiting NLRP3 (Fig. 4D–F). As shown in Fig. 4G, in the NC and OE microglia, HAX-1 and NLRP3 appeared to be co-localized in the cytoplasm without NLRP3 and ASC assembling. After OGD/R modeling, NLRP3 and ASC assembled to form the inflammasome structure, and this trend was decreased when HAX-1 overexpressed (Fig. 4G and S-Fig. 4C). These results indicated that HAX-1 inhibited the binding of ASC and NLRP3 in microglia and further reducing the formation of the inflammasome.

### HAX-1 regulated the formation of the NLRP3 inflammasome in the cerebral infarction zone in the mouse model of tMCAO by trimming the combination of ASC to NLRP3

**Fig. 5:**
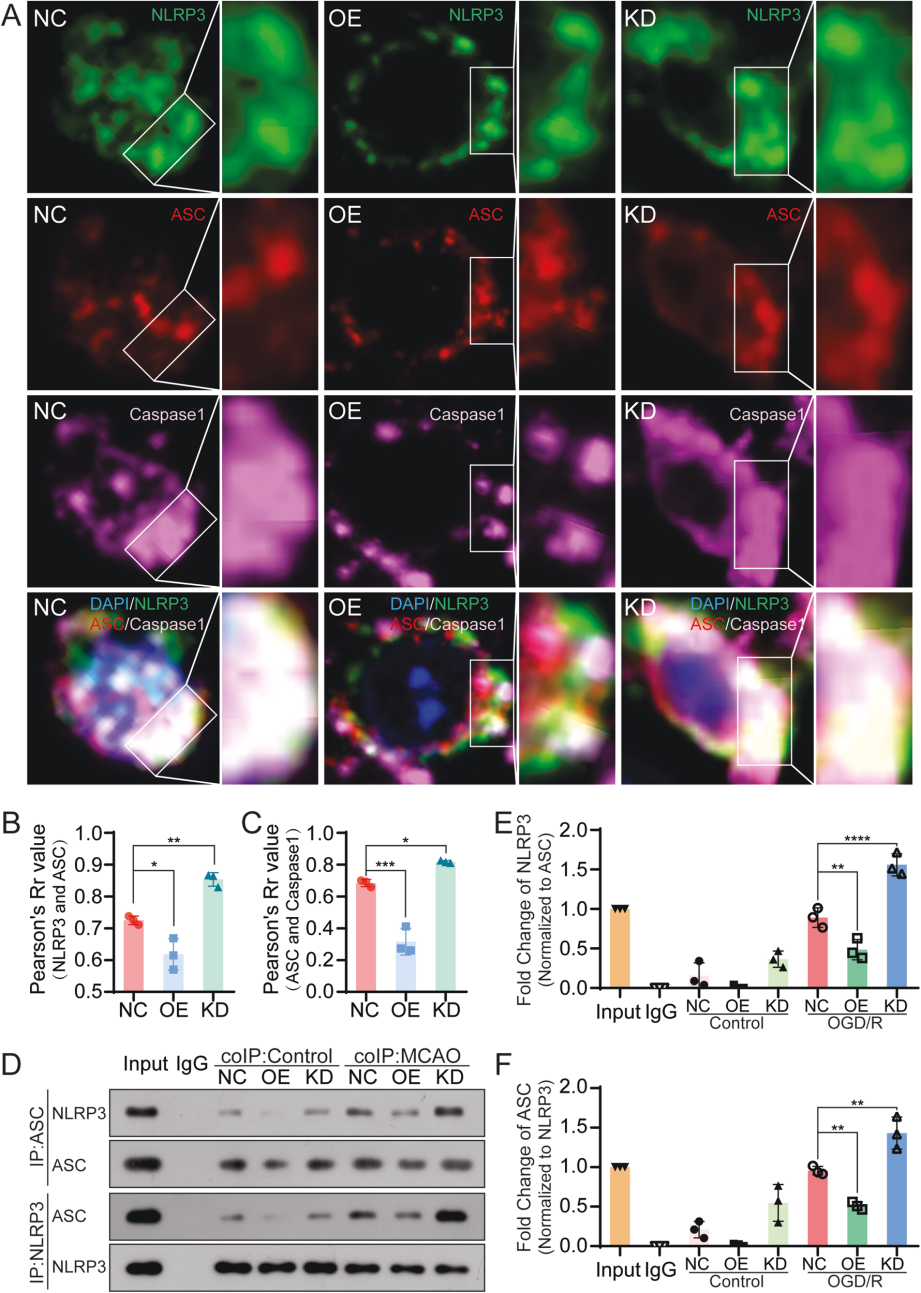
HAX-1 regulated the formation of the NLRP3 inflammasome in a mouse model of tMCAO. **A**–**C** Confocal microscopy revealed the colocalization of NLRP3, ASC, and Caspase1. Nuclei were stained with blue fluorescence, NLRP3 was stained with green fluorescence, ASC was stained with red fluorescence, and Caspase 1 was stained with purple fluorescence (original magnification: ×1000). **D**–**F** Co-immunoprecipitation (Co-IP) showed that HAX-1 affected the interaction between NLRP3 and ASC in tissue. *p < 0.05; **p < 0.01, ***p < 0.001, ****p < 0.0001.

To further study the effect of HAX-1 in regulating the formation of NLRP3 inflammasome, we performed Co-IP and immunofluorescent colocalization assays in a mouse model of tMCAO. As shown in Fig. 5A, in the NC group, NLRP3, ASC, and Caspase1 appeared to colocalize in the cytoplasm and aggregate into large fluorescent clumps. The knockdown of HAX-1 enhanced these features, while the overexpression of HAX-1 led to a reduction in the colocalization of NLRP3, ASC, and Caspase1. This colocalization pattern was also altered in a diffusely distributed punctate pattern in the cytoplasm (Fig. 5A–C). Co-IP showed that, compared with the control groups, the interaction of ASC and NLRP3 was clearly increased in the tMCAO group. In detail, NLRP3 increased by 1.49-fold in the HAX-1 KD group and decreased by 0.535-fold in the HAX-1 OE group compared with the NC group. The same trend was observed with baiting NLRP3 (Fig. 5D–F). These results indicated that HAX-1 inhibited the binding of ASC and NLRP3 and that this might inhibit the formation of the inflammasome. These results confirmed the function of HAX-1 in regulating the formation of the NLRP3 inflammasome in vivo.

### HAX1 regulated the formation of the inflammasome through competitive binding to bind NLRP3 protein with ASC

**Fig. 6:**
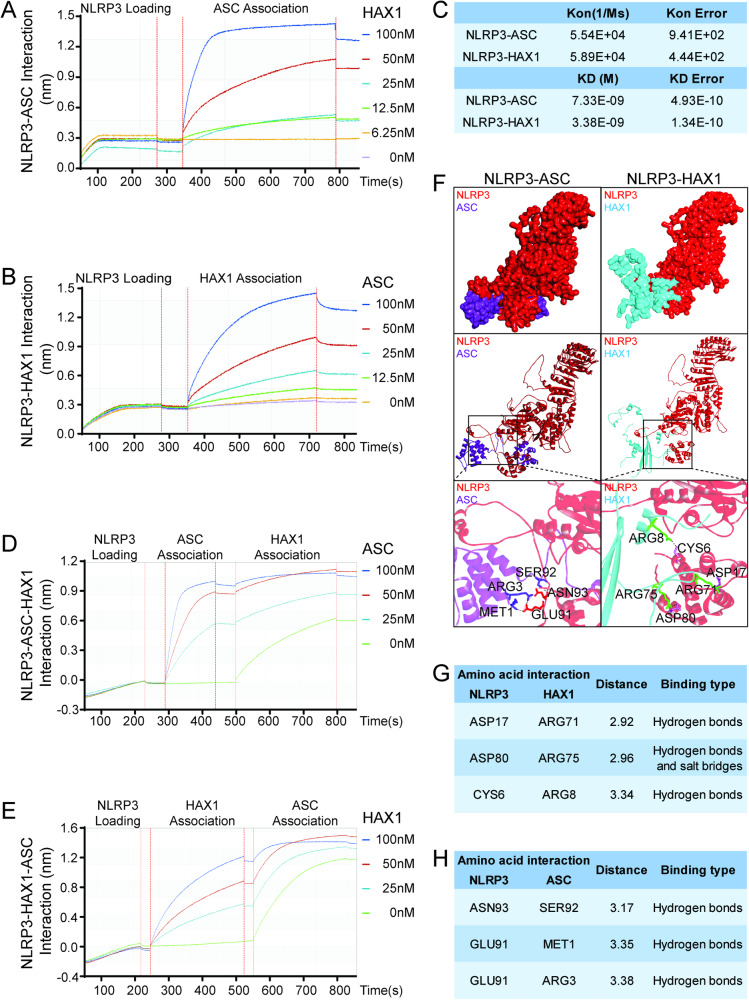
HAX1 regulated the formation of the inflammasome through competitive binding to bind NLRP3 protein with ASC. **A**–**C** BLI assay indicated the combination among HAX-1, ASC, NLRP3. **D** and **E** BLI assay tested the competitive binding of HAX-1 and ASC to NLRP3. **F**–**H** Molecular docking simulates NLRP3–ASC–HAX1 interaction. NLRP3 was labeled with red and HAX-1 was labeled with blue, ASC labeled with purple.

In order to further clarify the specific mechanism of HAX-1 inhibiting the binding of NLRP3 and ASC, Biolayer interferometry assays (BLI) were performed to verify the interaction of NLRP3, HAX-1, and ASC. First, we detected the combination of NLRP3-HAX-1 and NLRP3-ASC separately, and results showed both HAX-1 and ASC could bind to NLRP3 (Fig. 6A–C). The Kon of ASC-NLRP3 was 5.54E + 4, which was similar to the Kon of HAX-1-NLRP3 (5.89E + 4), and this means HAX-1 has a similar degree of binding with NLRP3 to ASC. And we also examined the combination between ASC and HAX-1, The result shows that there is no binding between HAX-1 and ASC (S-Fig. 6A). We then performed the HAX1–ASC–NLRP3 competitive binding test, and the results showed whether HAX-1 or ASC binds to NLRP3, the subsequent binding speed and degree of another protein are inhibited, and this effect shows a significant concentration dependence (Fig. 6E, F and S-Fig. 6B, C). These results indicate that HAX-1 and ASC have competitive binding with NLRP3, which explains the inhibitory effect of HAX-1 on the formation of inflammasome to a certain extent. Then, we used Discovery Studio to simulate the NLRP3–ASC–HAX1 interaction. As shown in Fig. 6H and I, HAX-1 and ASC have similar binding sites on NLRP3 protein, and the free energy of interaction between HAX1 and NLRP3 was −8.2 kcal/mol, which was similar to the free energy of ASC −8.7 kcal/mol (Fig. 6F–H). These results confirmed that HAX-1 can competitively bind to NLRP3 protein with ASC, thus inhibiting the formation of inflammasome.

## Discussion

Our study indicated that the levels of pyroptosis in microglia increased in a mouse model of tMCAO, and this change promoted apoptosis in nerve cells in the infarct area. At the same time, HAX-1 expression was found to be reduced in cerebral infarction. Based on the above findings, we established HAX-1 overexpressed and KD mice and microglia cell culture and used them to explore the role of HAX-1 in the pyroptosis of microglia and nerve cell apoptosis in the cerebral infarcted area. Following HAX-1 KD, there was an increase in pyroptosis in the microglia in both the tMCAO and OGD/R models; which was accompanied by the over-activation of the Caspase1/Gasdermin-D axis. Not surprisingly, the overexpression of HAX-1 led to the opposite results. Accordingly, IL-1 and IL-18 production was increased in HAX-1 KD microglia after OGD/R modeling, and nerve cell apoptosis increased in the tMCAO model these effects were inhibited by HAX-1 overexpression. We further investigated the formation of the NLRP3 inflammasome and found that HAX-1 KD markedly enhanced the assembly of NLRP3 and ASC after the I/R injury. A significant decline in such activity was observed in the HAX-1 overexpression group. Thereafter, we detected the binding of HAX-1-NLRP3 and ASC-NLRP3 proteins, which showed that HAX-1 and ASC had competitive binding with NLRP3. Our results show that HAX-1 could inhibit the binding of these two proteins.

Pyroptosis is a form of programmed cell death and is characterized by the formation of cell membrane pores and the release of inflammatory factors such as IL-1 and IL-18; these cellular changes eventually lead to cell death and inflammatory damage to the surrounding cells [12, 13]. Microglia are innate immune effector cells in the central nervous system, and the inflammatory injury caused by microglia pyroptosis has been reported to be related to a variety of diseases in the nervous system [14]. To further study the correlation between cerebral ischemia-reperfusion injury and microglia pyroptosis, a mouse model of tMCAO was utilized, and the data demonstrated an increased level of apoptosis in nerve cells accompanied by a higher level activation of Caspase1 and Gasdermin-D in the cerebral ischemic area. The Caspase1 inhibitor, belnacasan, alleviated nerve cell apoptosis, suggesting that the inflammatory injury caused by pyroptosis in the cerebral ischemic area led to increased levels of apoptosis in nerve cells. This finding was consistent with recent research results on pyroptosis in cerebral I/R injury [30, 31]. The data of our immunofluorescence staining showed that the inflammasome in cerebral ischemic areas was mainly formed in the microglia, thus indicating that microglia were the main effector of inflammatory injury in cerebral I/R injury. Therefore, the investigation of the mechanisms underlying microglial pyroptosis may provide new targets for treating cerebral I/R injury.

HAX-1, as an axiomatic pro-survival protein, was first discovered in congenital severe neutropenia and was shown to inhibit apoptosis in nervous cells [16]. Recent research has shown that HAX-1 was involved in the regulation of neuronal synapse formation and ion channel activities and played a neuroprotective role [20]. In our previous studies, we found that HAX-1 promoted cellular proliferation and attenuated apoptosis in the conditions of tumor-glioblastoma in the central nervous system [32]. Therefore, we hypothesize that HAX-1 may also play a similar neuroprotective role in cerebral I/R injury. Initially, we detected the expression of HAX-1 in the mouse model of tMCAO and found that the expression of HAX-1 decreased after cerebral ischemia. Then, we manipulated the expression of HAX-1 in the experimental mouse brain by AAV transfection. We found that after HAX-1 overexpression, neural cell apoptosis was reduced, and the activation of Caspase1 and Gasdermin-D in cerebral ischemic tissue was also significantly reduced. On the contrary, HAX-1 KD showed the opposite results. Moreover, in the absence of HAX-1 protein in brain tissue, the severity of cerebral infarction increased, and the nervous cell apoptosis also increased significantly. Both Caspase1 and Gasdermin-D are known to be the key effector proteins of pyroptosis and are able to induce cell membrane perforation and the subsequent release of inflammatory mediators; these effects lead to apoptosis in the surrounding cells. Our results indicated that HAX-1 played a protective role in cerebral I/R injury through the inhibition of nervous cell apoptosis in the surrounding areas adjacent to infarction, which was caused by a pyroptosis-induced inflammatory storm. Immunofluorescence study showed that the formation of the NLRP3 inflammasome in microglia in the ischemic area decreased after the overexpression of HAX-1, while the formation of the inflammasome was enhanced following HAX-1 KD. The above findings also suggested that HAX-1 played a critical role in neuroprotection following cerebral I/R injury, likely through inhibition of microglial pyroptosis.

Next, we further investigated the effect of HAX-1 in vitro. First, we simulated cerebral I/R injury on microglia BV2 cells in a glucose oxygen deprivation/reoxygenation model and observed the changes in HAX-1 expression. Consistent with the results obtained in vivo, the expression of HAX-1 in microglia decreased after OGD/R modeling. Then, we manipulated the expression of HAX-1 in microglia by adenovirus transfection and observed the effect of HAX-1 on microglial pyroptosis. Correlated with our in vivo results, the overexpression of HAX-1 inhibited the formation of the NLRP3 inflammasome, decreased the activation of caspase-1 and gasdermin-D and reduced the release of IL-1 and 18 in microglia. On the contrary, HAX-1 KD revealed the opposite results in the same experimental designs. These findings indicate that HAX-1 influences the pyroptosis of microglia. To ensure the role of NLRP3 in HAX-1 protective effects, we used the selective NLRP3 inhibitor MCC950 for further research [33]. MCC950 blocked the activation of Caspase 1 and Gasdermin-D induced by HAX-1 expression to a certain level; this finding again approved the role of NLRP3 in the HAX-1-mediation of microglial pyroptosis.

Hsp90, Arp2/3, and cortactin proteins have been reported to bind to HAX-1, which was important for HAX-1 to reveal neuroprotective function [20, 21]. However, there is no direct evidence for the correlation between these proteins and pyroptosis, and HAX-1 may inhibit pyroptosis through other molecular mechanisms. We found that HAX-1 could inhibit microglial pyroptosis and NLRP3 inflammasome formation. NLRP3/ASC inflammasome, thus enhancing the process of pyroptosis [34], may play an important role in the inhibition of pyroptosis by HAX-1. So, we investigated the relationship between HAX-1 and NLRP3 inflammasome. Co-IP assays were performed to verify the interaction of HAX-1 and NLRP3, which declined after OGD/R modeling. Therefore, promoting interaction between HAX-1 and NLRP3 may represent a key breakthrough in the determination of the mechanisms through which HAX-1 can influence microglial pyroptosis. Based on that hypothesis, we performed Co-IP and immunofluorescence colocalization to detect the binding of ASC to NLRP3 protein and the formation of the NLRP3 inflammasome under different expression levels of HAX-1. Compared with the control, the overexpression of HAX-1 definitely reduced the combination of NLRP3 and ASC; however, this combination was enhanced in the KD group. Immunofluorescence co-localization staining showed that the degree of CO localization of HAX-1 and NLRP3 was significantly weakened after OGD/R modeling, with the enhancement of NLRP3-ASC co-localization. HAX-1 overexpression reduced the co-localization enhancement of NLRP3 and ASC after modeling to a certain extent. These results indicated that HAX-1 could interfere with the assembly of NLRP3-ASC inflammasome. The in vivo data were also consistent with data derived from the in vitro experiments, particularly in regard to colocalization imaging. HAX-1 KD significantly enhanced the colocalization of NLRP3, ASC, and Caspase1 in the cytoplasm, thus forming an obvious inflammasome-like structure, as reported previously [35, 36]. The overexpression of HAX-1 attenuated such changes; as most of the fluorescence signals of the three proteins were presented scattered in a diffuse and punctate pattern throughout the cells. These findings again approved the role of NLRP3 in the HAX-1-mediation of microglial pyroptosis.

NLRP3 inflammasomes are common types in inflammatory pathways, among which NLRP3-ASC inflammasome is one of the classic forms, which plays an important role in activating downstream pyroptosis pathways in various tissues [37–39]. In previous studies, we found that HAX-1 can bind to NLRP3 and interfere with the assembly of NLRP3-ASC inflammasomes. To further explore the specific intervention mode of HAX-1 on NLRP3-ASC inflammasomes, we performed BLI assays, and results show that NLRP3 could bind to both HAX-1 and ASC, while there was no combination between HAX-1 and ASC. Sequential binding assays showed HAX-1 could inhibit the binding of NLRP3 and ASC in a concentration-dependent manner, while ASC could also reverse the binding of HAX-1 and NLRP3 in the same way. These results indicated that there was competition between HAX-1 and ASC to bind to NLRP3. Recent studies have determined that activated NLRP3 recruits and binds with ASC through PYD–PYD interactions [40, 41]. Therefore, we speculate that HAX-1 may inhibit the assembly of NLRP3 and ASC through binding to the PYD domain of NLRP3 protein. Therefore, we preliminarily verified this hypothesis through protein molecular docking simulation. The results showed that in the NLRP3 PYD domain, HAX-1 protein and ASC had similar binding domains and binding free energy. These results explain the mechanism of the effect of HAX-1 on NLRP3-ASC inflammasome to a certain extent.

**Fig. 7:**
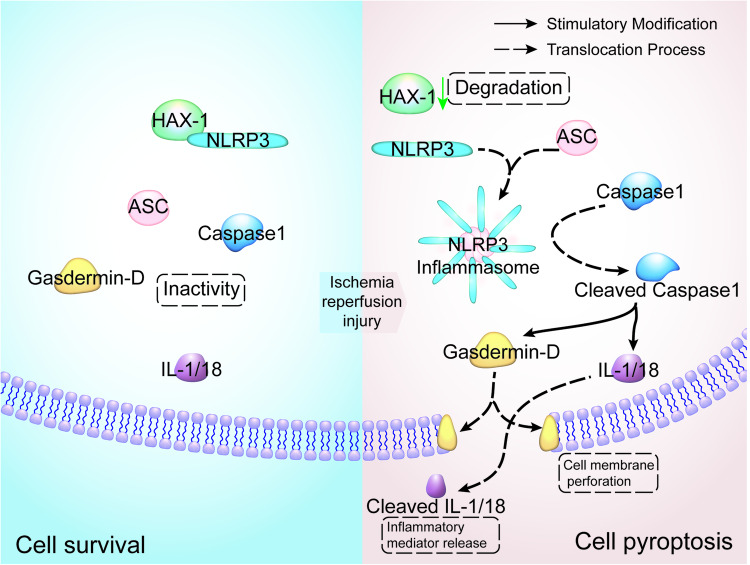
Schematic diagram of HAX1 inhibiting microglial pyroptosis. Schematic diagram depicted the mechanism underlying the inhibition of HAX-1 on microglia pyroptosis.

In summary, our study showed HAX-1 attenuated microglial pyroptosis both in vivo and in vitro and reduced the inflammatory responses in cerebral infarction through inhibition of proinflammatory cytokines such as IL-1 and IL-18. Such anti-pyroptosis effect appears to be partially mediated by the inhibition of the interaction between ASC and NLRP3 by competitive binding and the subsequent attenuation of NLRP3 inflammasome formation (Fig. 7). However, the underlying mechanisms regarding the effects of HAX-1 in cerebral I/R injury are still unclear and will be further investigated in our future studies. In addition, the role and mechanism of HAX-1 in neuronal apoptosis will also be considered with the aim of identifying possible therapeutic targets for HAX-1 in cerebral I/R injury in future research.

## Conclusion

In the present study, we demonstrated for the first time that HAX-1 can inhibit microglial pyroptosis and the production of inflammatory cytokines and also attenuates NLRP3 inflammasome formation through competitive binding to NLRP3 with ASC.

## Methods

All experimental procedures were approved by the Care of Experimental Animals Committee of Zhengzhou University and were performed in accordance with current guidelines and regulations.

### Antibody and reagents

The following primary antibodies were used for Western blot, co-IP, and immunofluorescence: anti-HAX-1 antibody (BD, cat: 610825), anti-NLRP3 antibody (CST, cat: 15101), anti-ASC antibody (CST, cat: 67824), anti-Caspase1 antibody (CST, cat: 24232), anti-Cleaved Caspase1 antibody (CST, cat: 89332), anti-GSDMD antibody (CST, cat:93709), anti-TMEM119 antibody (Abcam, cat: ab209064), anti-β-actin antibody (Abmart, cat: P30002S), Goat anti-rabbit antibody HRP linked (CST, cat: 7074), Goat anti-mouse antibody (CST, cat:7076). The co-IP assays were performed by using Pierce Crosslink Magnetic IP Kit (Thermo Scientific, Cat: 88805). The immunofluorescence assays were performed by using Opal 7-color manual IHC kit (PerkinElmer, NEL801001KT). The following recombinant proteins were used for BLI assay: recombinant NLRP3 (Abmart, cat: EHH22785), recombinant ASC (Proteintech, cat: Ag28424), recombinant HAX-1 (Proteintech, cat: Ag27244). NLRP3 selective inhibitor MCC950 was purchased from APExBIO company, cat: C3780.

### Animals and adeno-associated virus transfection

Healthy male C57BL/6 mice (8–10 weeks of age, weighing 20–30 g) were obtained from the Animal Center of Zhengzhou University. The mice were raised in a humidity-controlled room at 25 ± 2 °C under a 12:12-h light/dark cycle with free access to food and water. Mice were randomized according to the random number table, but not blinded.

To manipulate the expression of HAX-1 in the brains of experimental mice, we delivered a HAX-1 overexpression adeno-associated virus (AAV) containing the HAX-1 coding sequence (CDS), a HAX-1 knocked down AAV containing a HAX-1 small interfering RNA (siRNA) sequence, and a control AAV containing the empty plasmid only. All viruses were constructed and packaged by Shanghai GeneChem. We individually delivered the different AAVs at 1 × 10^9^ vg into the lateral ventricles of mice by stereotactic injection to generate a HAX-1 overexpression group (OE group), a HAX-1 knockdown group (KD group), and a negative control group (NC group) and each group included 24 mice. The virus dose was according to the recommended dose of Shanghai GeneChem, and was verified in the pre-experiment.

The siRNA sequence was designed as follows:

accggGCCCATGACAGTTCCAGAAttcaagagaTTCTGGAACTGTCATGGGCttttt

### Generation of the transient middle cerebral artery occlusion (tMCAO) model

C57BL/6 mice (20–30 g) were continuously anesthetized with isoflurane during operation. Then, a midline longitudinal incision was created in the neck to expose the right common carotid artery. The right external carotid arteries were ligated, and then a monofilament was slowly inserted into the artery until a slight resistance was felt; this indicated that the filament had blocked the blood flow to the middle cerebral artery. The filament was held in place for 2 h and then withdrawn for reperfusion. Paralysis in the left foreleg or a circular motion demonstrated that the tMCAO model had been generated successfully. A sham-operation group was also created using the same procedures as the tMCAO group but without the insertion of the monofilament and reperfusion. 24 WT mice were randomly divided into two groups for tMCAO model or sham operation by random number table for exploring the correlation between microglial pyroptosis and peripheral nerve injury in cerebral ischemia-reperfusion injury. AAV intervention mice were also randomly divided into two groups for the tMCAO model and sham operation for subsequent trials to verify the effect of HAX-1.

### Cell culture and adenovirus transfection

BV2 cells were obtained from the Cell Bank of the Chinese Academy of Sciences, cultured in Dulbecco’s modified eagle medium (DMEM)/high glucose medium (DMEM) (Coning, USA) with 10% fetal bovine serum (FBS, Gibco) at 37 °C in a humidified atmosphere containing 5% CO_2_ and 95% air.

In order to manipulate the expression of HAX-1 in BV2 cells, we delivered adenoviruses containing the HAX-1 CDS sequence or a HAX-1 siRNA sequence. The negative control virus carried the empty plasmid only. All viruses were constructed and packed by Shanghai GeneChem. The efficiency of gene transfer was evaluated by the expression of GFP. Over 90% of BV2 cells were infected at 500 MOI on the second day after infection. The siRNA sequence was designed as follows:

siRNA 1: cgGCAACTTTGGCTTTGATGA

siRNA 2: ccAGCCCAAATCGTATTTCAA

The infected BV2 cells were washed with PBS and harvested for western blotting or used in other experiments.

### 2,3,5-Triphenyltetrazolium chloride (TTC) staining

Twelve hours after reperfusion, the experimental mice were decapitated. The brains were removed and placed in ice-cold saline for 5 min. Then, the tissues were sliced into coronal sections and incubated in a 2% TTC solution for 30 min at 37 °C. The normal cerebral parenchyma was stained red, while infarcted tissues remained unstained. Brain lesion volume was calculated by multiplying the mean slice thickness by the sum of infarction areas in a total of five sections and analyzed using Image-J (Media Cybernetics, Bethesda, MD, USA). TTC staining included three individual biological replicates in every group.

### Hematoxylin–eosin (H&E) staining

After 12 h of reperfusion, we performed H & E staining for histological analyses. The animals were anesthetized and perfused trans-cardially. The brain and dura mater were removed and fixed in a 4% paraformaldehyde solution. After paraffin embedding, 2-μm-thick coronal sections were created and stained with H&E. The H&E staining included three individual biological replicates in every group.

### Generation of an oxygen–glucose deprivation/reoxygenation (OGD/R) model and drug administration

Next, we generated an oxygen–glucose deprivation and reoxygenation (OGD/R) model, an in vitro model that mimics in vivo cerebral ischemia-reperfusion injury [42]. The OGD/R model was established as described below. The cell culture medium of the BV2 cells was replaced with glucose-free DMEM medium (Solarbio, China) containing 5 mM ATP and placed into a hypoxic incubator that contained mixed gas (85% nitrogen/10% hydrogen/5% carbon dioxide) for 2 h at 37 °C. Then, the medium was replaced with DMEM medium containing 10% FBS and placed in a humidified atmosphere containing 5% CO_2_ and 95% air at 37 °C for 2 h. After establishing the OGD/R model, we collected cells for subsequent experiments.

### Immunofluorescence and confocal microscopy

Cells were washed with PBS twice and fixed in 4% paraformaldehyde for 12 h at 4 °C. Immunofluorescent staining was then carried out with Opal 7-color IHC Kits in accordance with the manufacturer’s instructions (PerkinElmer, USA). We detected TMEM119, HAX-1, NLRP3, ASC, pIRF3 by applying a range of primary antibodies (TMEM119 from Abcam, USA; NLRP3, ASC, pIRF3 from CST, USA; HAX-1 from BD Biosciences) and staining with different fluorescent dyes. Laser scanning confocal microscopy (Nikon) was used for cell observation. Three individual biological replicates were performed in immunofluorescence for both tissue and cell samples.

### Enzyme-linked immunosorbent assays (ELISAs)

Following OGD/M modeling, we collected serum-free medium for ELISAs. Mouse IL-1, IL-18, IL-6, and IFNβ ELISA kits (Shanghai Duma Biotechnology Co.) were used in accordance with the manufacturer’s instructions. Three individual replicates in every group were contained in ELISA assays.

### The detection of apoptosis by TUNEL assays

After dewaxing, the tissue sections were washed with PBS and incubated with protein K at 37 °C for 30 min. Then, the following steps were carried out in sequence, and the sections were washed three times with PBS at the end of each step. First, sections were incubated with DNaseI reaction solution at 37 °C for 30 min and then with equalization buffer and biotin-11-dUtp and TDT enzyme at 37 °C in the dark for 30 min. Next, sections were incubated with labeling buffer containing streptavidin TRITC at 37 °C in the dark for 30 min. Finally, the nuclei were counterstained with DAPI, and the sections were photographed and analyzed by fluorescence microscopy. Three individual replicates in every group were contained in TUNEL assays.

### Co-Immunoprecipitation (Co-IP)

Cells were homogenized in cell lysis buffer (Solarbio) supplemented with 1 mM phenylmethylsulfonyl fluoride (PMSF) and a complete protease inhibitor mixture (Solarbio). The homogenate was then centrifuged at 11,000 rpm for 15 min at 4 °C. Next, the supernatant was incubated with ASC/HAX-1/NLRP3 antibody or rabbit/mouse normal IgG crosslinked beads at 4 °C for 1 h with rotation. The pretreatment of beads and the immunoprecipitation steps were carried out with a Pierce Crosslink Magnetic IP Kit in accordance with the manufacturer’s instructions. Proteins were identified by western blotting or protein mass spectrometry. Homogenates from tissue or cells were used as positive controls. Three individual replicates in every group were contained in Co-IP assays.

### Western blotting

Cells were collected and lysed in cell lysis buffer (Solarbio) for 30 min on ice and then centrifuged at 12,000 rpm (15 min, 4 °C). The supernatant was collected and the concentration of total protein was determined with a bicinchoninic acid protein assay (BCA)Kit (Solarbio). The samples were mixed with 5× SDS loading buffer (Solarbio) and boiled at 100 °C for 10 min. An equal amount of each sample was then separated by SDS–PAGE and the proteins were transferred onto a polyvinylidene difluoride membrane. The membranes were then incubated with 5% BSA at room temperature for 1–2 h, followed by primary antibodies (mouse-anti-human HAX-1 from BD; from Proteintech; Rabbit anti-mouse NLRP3, ASC, Caspase1, Cleaved Caspase1, GasderminD, Cleaved GasderminD, IKKε, pIKKε, TBK1 and pTBK1 from CST; β-actin from Bioworld Technology) incubation at 4 °C for 12 h. Membranes were then washed three times with TBST and incubated with HRP-conjugated secondary antibodies (ZSGF-BIO). Visualization was achieved using a SuperSignal West Pico Trial Kit (Thermo). Relative intensities of the bands were analyzed by Image-J software. Three individual biological replicates were performed in a Western blotting assay.

### Biolayer interferometry (BLI)

Recombinant full-length HAX-1 was purchased from Proteintech company. Recombinant full-length ASC and partial NLRP3^1-536aa^ were purchased from Abmart company. The binding affinities among recombinant NLRP3, HAX-1, and ASC were measured by BLI assay using the Octet RED96 system (ForteBio). All proteins were diluted in PBS. In brief, NLRP3 was biotinylated (10 mg/mL) and then immobilized onto SA Dip and Read biosensors (ForteBio) and balanced with PBS. The biosensors were then exposed to full-length HAX-1 (0, 6.25, 12.5, 25, 50, 100 nM) or ASC (0, 12.5, 25, 50, 100 nM), followed by washing (dissociation) with PBS. Binding affinities (*K*_on_ and *K*_d_) were calculated using the Data Analysis software (ForteBio). The binding affinities between HAX-1 and ASC were performed as the above process.

To analyze the sequential bindings of HAX-1 and ASC to NLRP3, NLRP3 (10 mg/mL) was immobilized onto SA Dip and Read biosensors and balanced with PBS. The loaded biosensors were first exposed to 25/50/100 nM HAX-1, balanced again with PBS, and then exposed to 100 nM ASC. Alternatively, the loaded biosensors were first exposed 25/50/100 nM ASC, balanced again with PBS, and then exposed to 100 nM HAX-1. All biosensors were then washed with PBS [43]. Three individual replicates were performed for each BLI assay.

### Protein–protein interaction prediction

The protein structure model adopts the published or UniProt system to provide the predicted structure. The molecular docking software Discovery Studio was used for simulation research. Import the structure into Discovery Studio, hydrogenate the protein, and give CHARMM force field for protein preparation. Then, the protein interaction prediction is completed by h-dock. Finally, the free energy is calculated in PDBePISA, which ranks in the top 10 according to the docking results. Referring to the binding domain reported by NLRP3 and ASC, the optimal interaction model is selected for mapping [44].

### Statistical analysis

Prism software (GraphPad 8.1) was used for all statistical analyses. Data were expressed as means ± standard deviation (SD). One-way analysis of variance (ANOVA) was used for comparisons among multiple groups, followed by ordinary one-way ANOVA for multiple comparisons, and a post-hoc test (Bonferroni) was employed. The unpaired Student’s *t*-test was used to compare the means of the two groups. The equal variances were checked prior to ensure the validity of the results. All the experiments were repeated at least 3 times. *t*-tests were two-sided, and the results were considered statistically significant at *p* < 0.05.

### Supplementary information

Supplement materials

## Data Availability

All data are available upon reasonable request. Please contact the corresponding author for any data inquiries.
