# Supplementary material for: HAX-1 interferes in assembly of NLRP3-ASC to block microglial pyroptosis in cerebral I/R injury
Source: Cell Death Discov. 2024 May 29;10:264. doi: 10.1038/s41420-024-02005-3 (PMC11136987; doi:10.1038/s41420-024-02005-3)

Supplementary Figures:

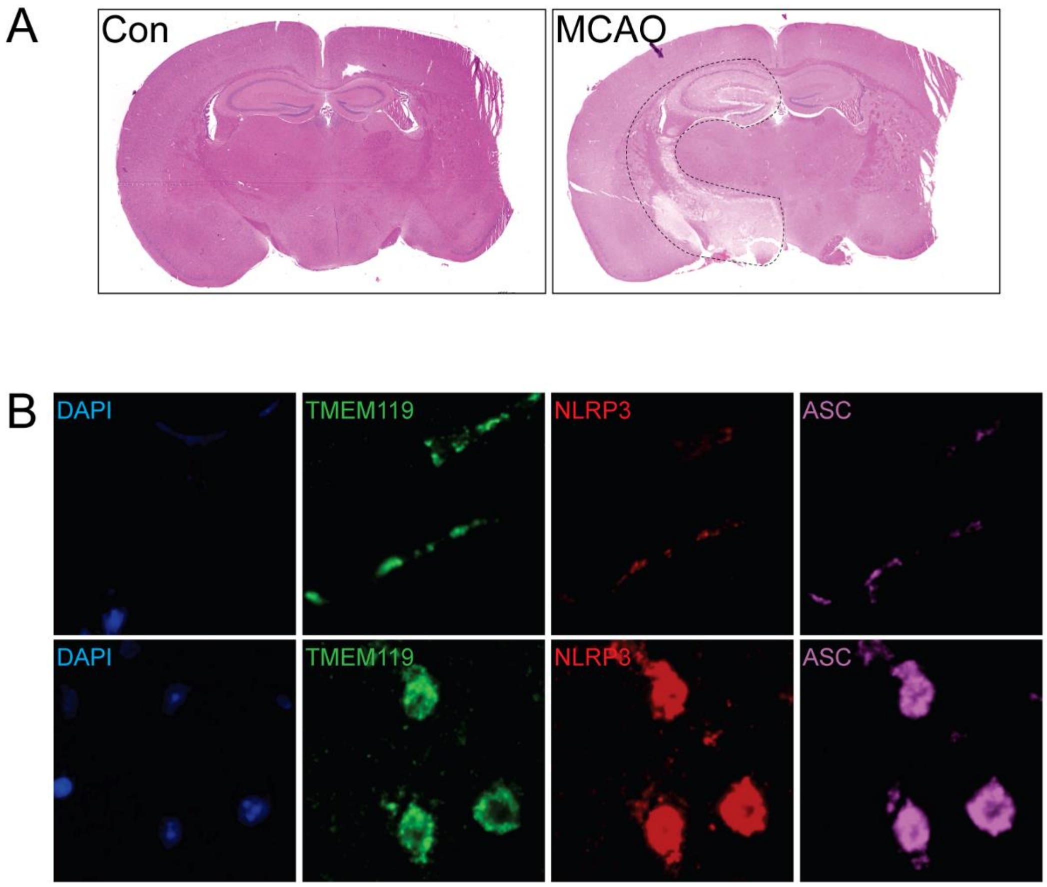

S-Figure 1. Microglial pyroptosis in the cerebral infarction area in a mouse model of tMCAO. (A) H&E staining of brain tissue from the MCAO and control animals, The infarct area was labeled with a dotted line. (B) Confocal microscopy revealed the colocalization of NLRP3 and ASC. NLRP3 was stained with red fluorescence, ASC was stained with purple fluorescence, TMEM119 was stained with green fluorescence, and nuclei were stained with blue fluorescence (magnification: 600×).

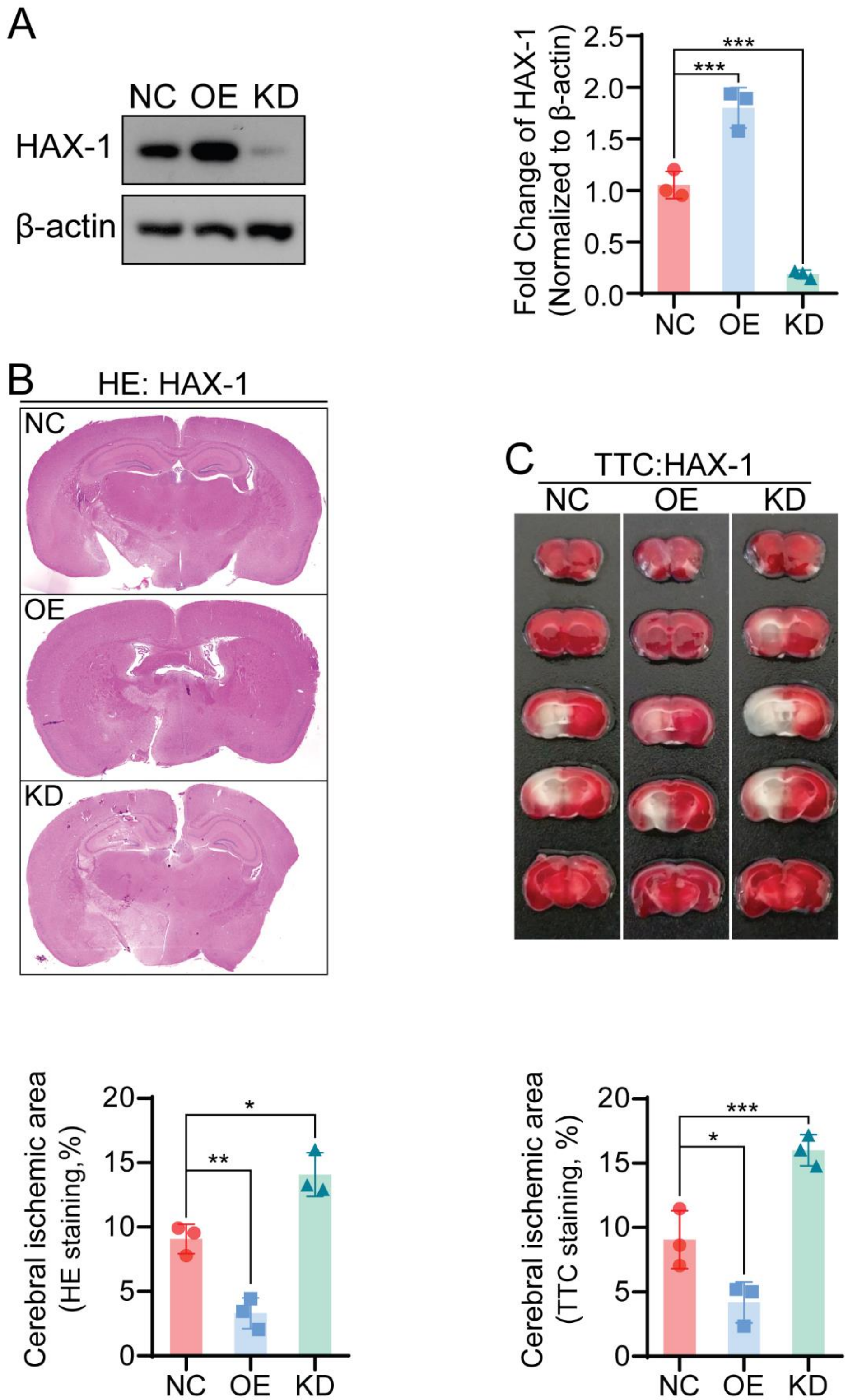

S-Figure 2. Expression levels of HAX-1 were reduced in the MCAO model and affected pyroptosis in microglia. (A) Western blotting was used to detect changes in HAX-1 expression in cerebral regions after transfection with AAVs. β-actin was used as a loading control. (B) H&E staining demonstrated the effects of HAX-1 on cerebral infarction in a mouse model of MCAO. (C) TTC staining revealed the effect of HAX-1 on cerebral infarction in a mouse model of MCAO.. *p<0.05; **p<0.01, ***p<0.001; ****p<0.0001.

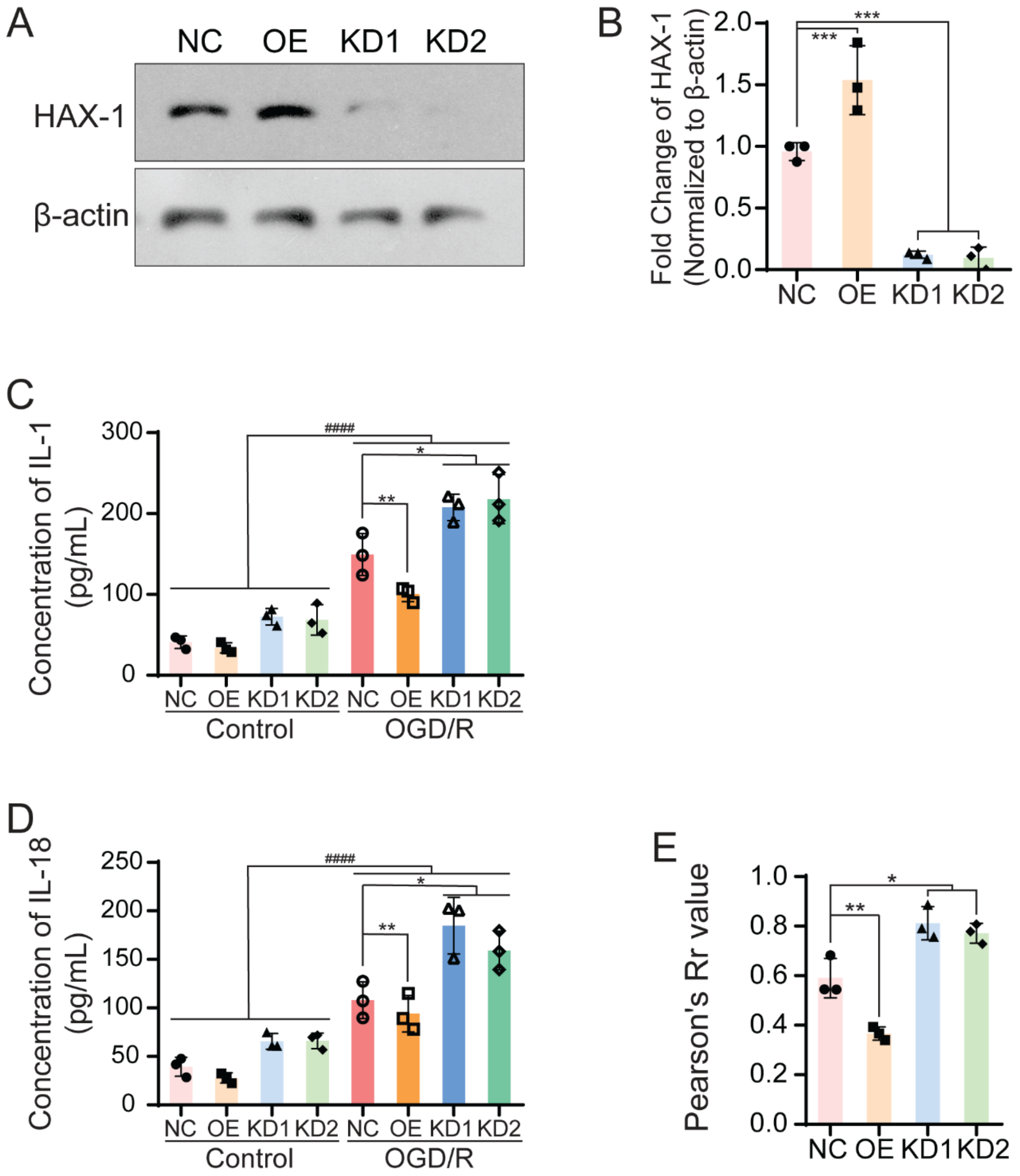

S-Figure 3. HAX-1 regulated the pyroptosis pathway and inflammasome formation in microglia. (A and B) Western blotting was used to detect alterations in HAX-1 expression in cerebral regions in response to AAVs. β-actin was used as a loading control. (C and D) ELISA assays indicated that HAX-1 altered the levels of IL-1 and IL-18. E. Pearson's Rr value of NLRP3-ASC in confocal immunofluorescence assays. *$p<0.05$; **$p<0.01$, ***$p<0.001$, $^{\#\#\#\#}p<0.0001$.

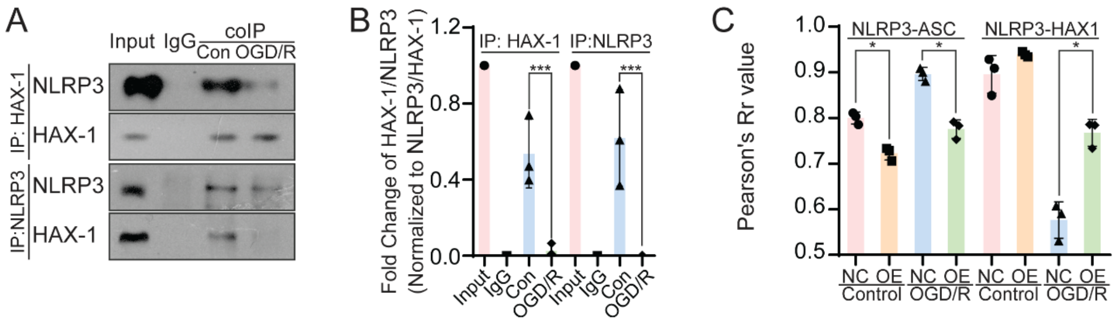

S-Figure 4. HAX-1 regulated inflammasome formation in microglia *via* NLRP3 i nflammasome. (A-B) Co-immunoprecipitation (Co-IP) showed that HAX-1 could interact with NLRP3 and that this interaction was attenuated after OGD/R. (C) Pearson's Rr value of NLRP3-ASC and NLRP3-HAX1 in confocal immunofluorescence assays. *p<0.05; ***p<0.001.

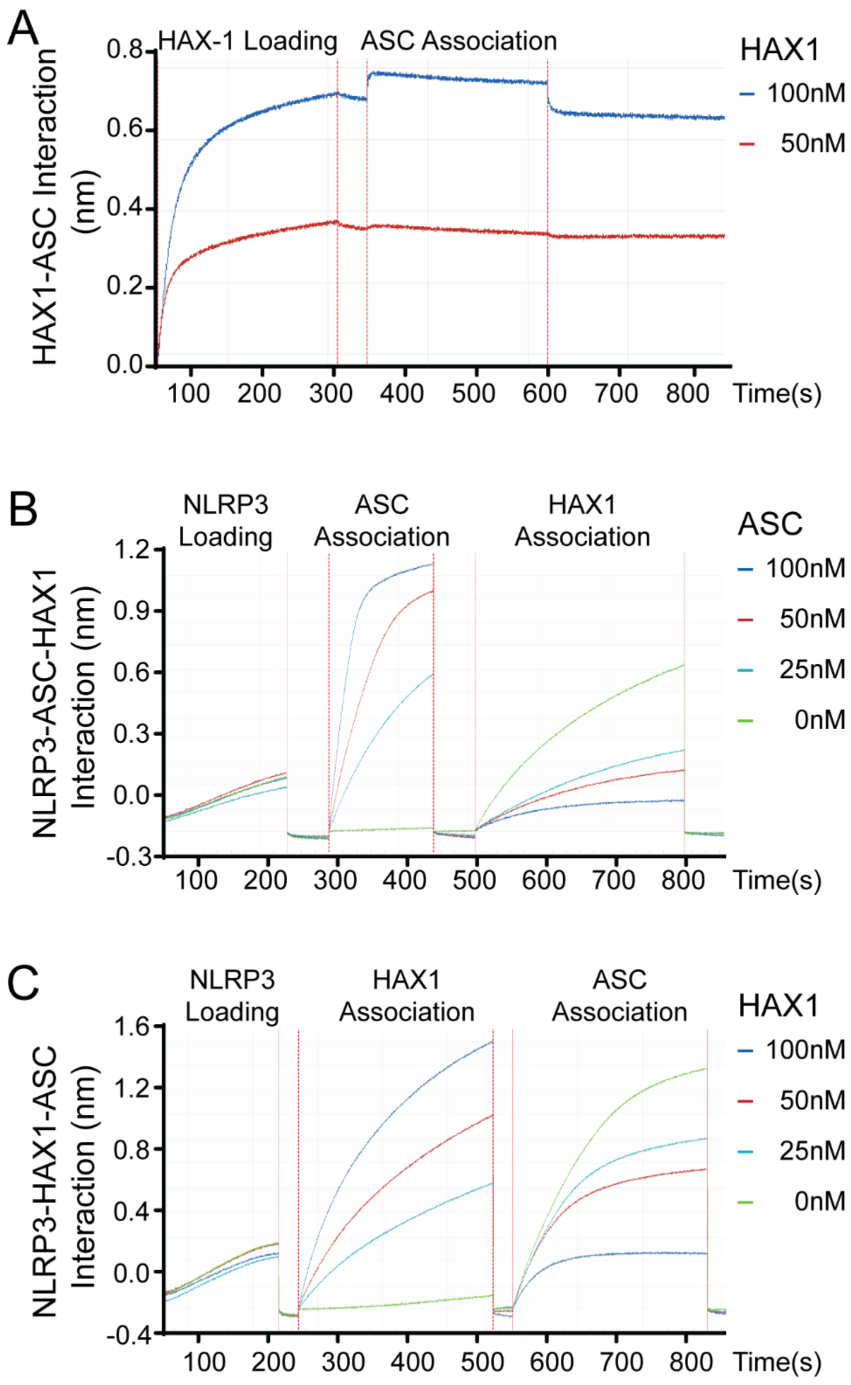

S-Figure 6. HAX1 regulated the formation of the inflammasome through competitive binding to bind NLRP3 protein with ASC. A. BLI assay indicated no combination between HAX-1 and ASC. (B and C) BLI assay tested the competitive binding of HAX-1 and ASC to NLRP3.

Western blot original bands

Figure 1 Supplement

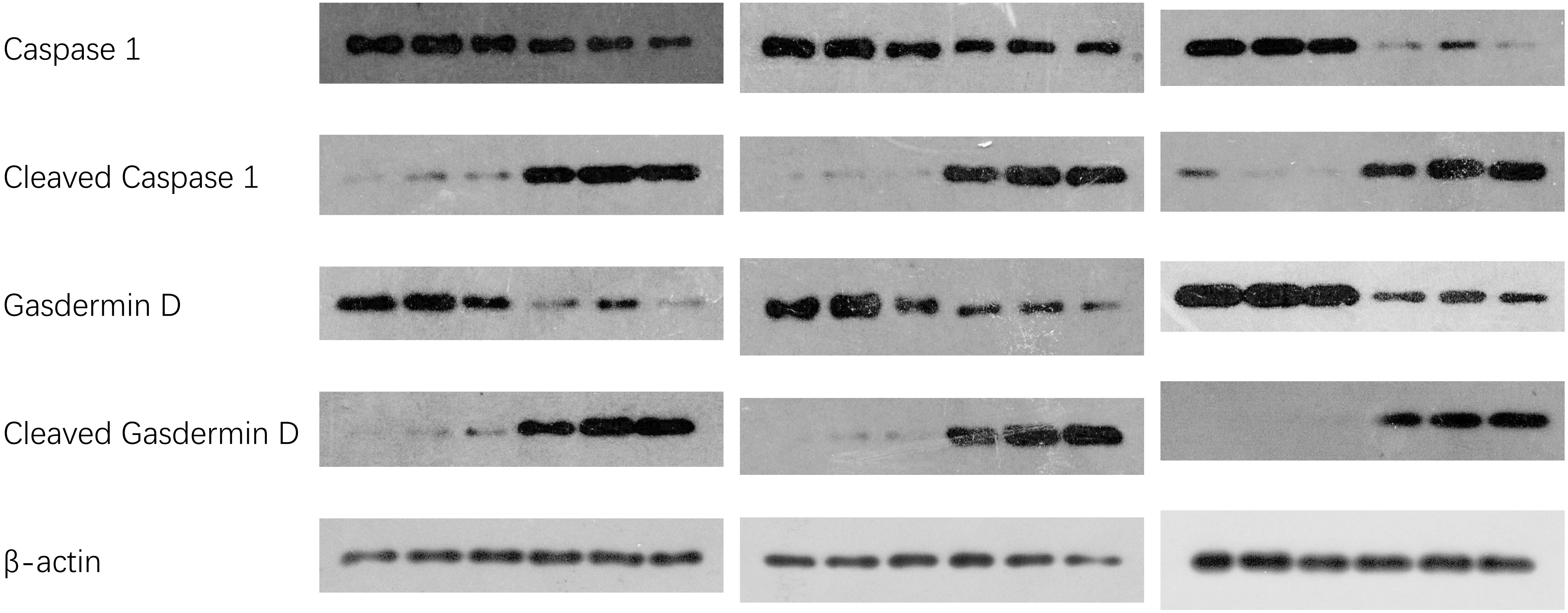

Figure 2 Supplement

Western blot—HAX-1 expression in MCAO

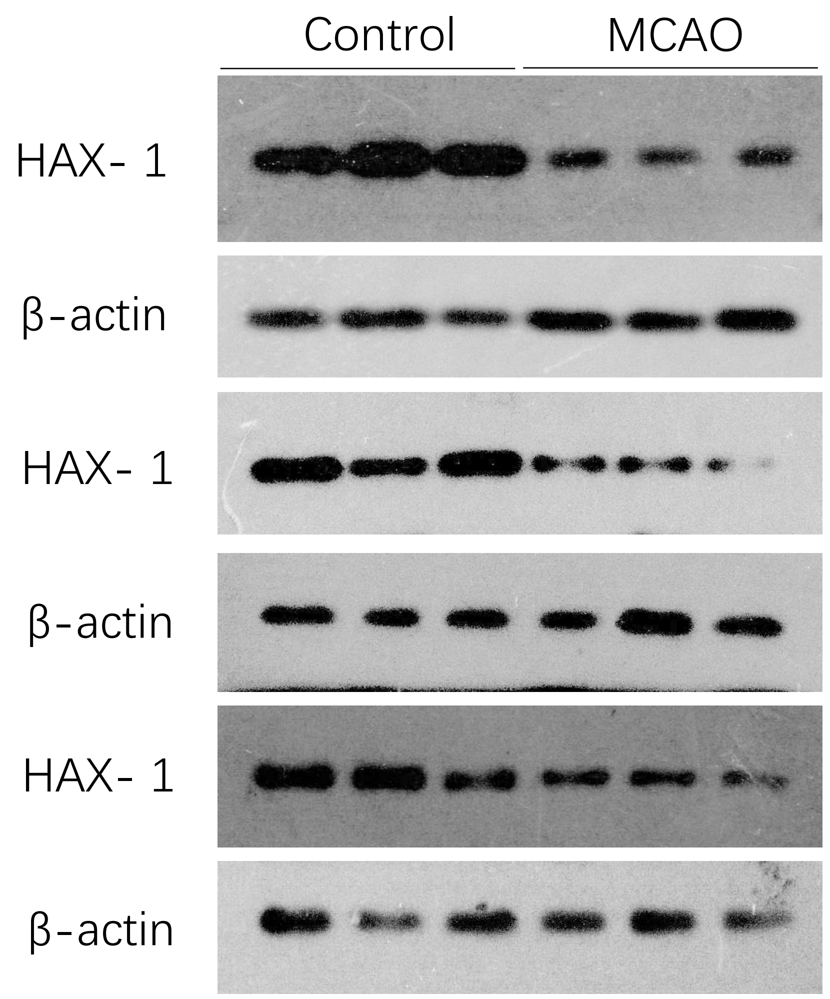

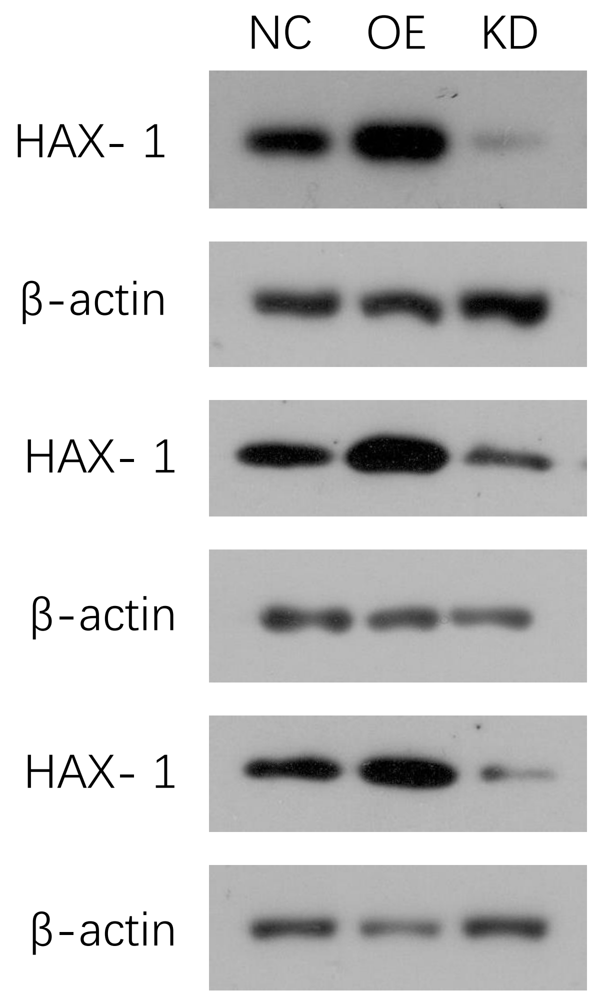

Figure 2 Supplement

Western blot—Activation of Caspase1/GasderminD in MCAO

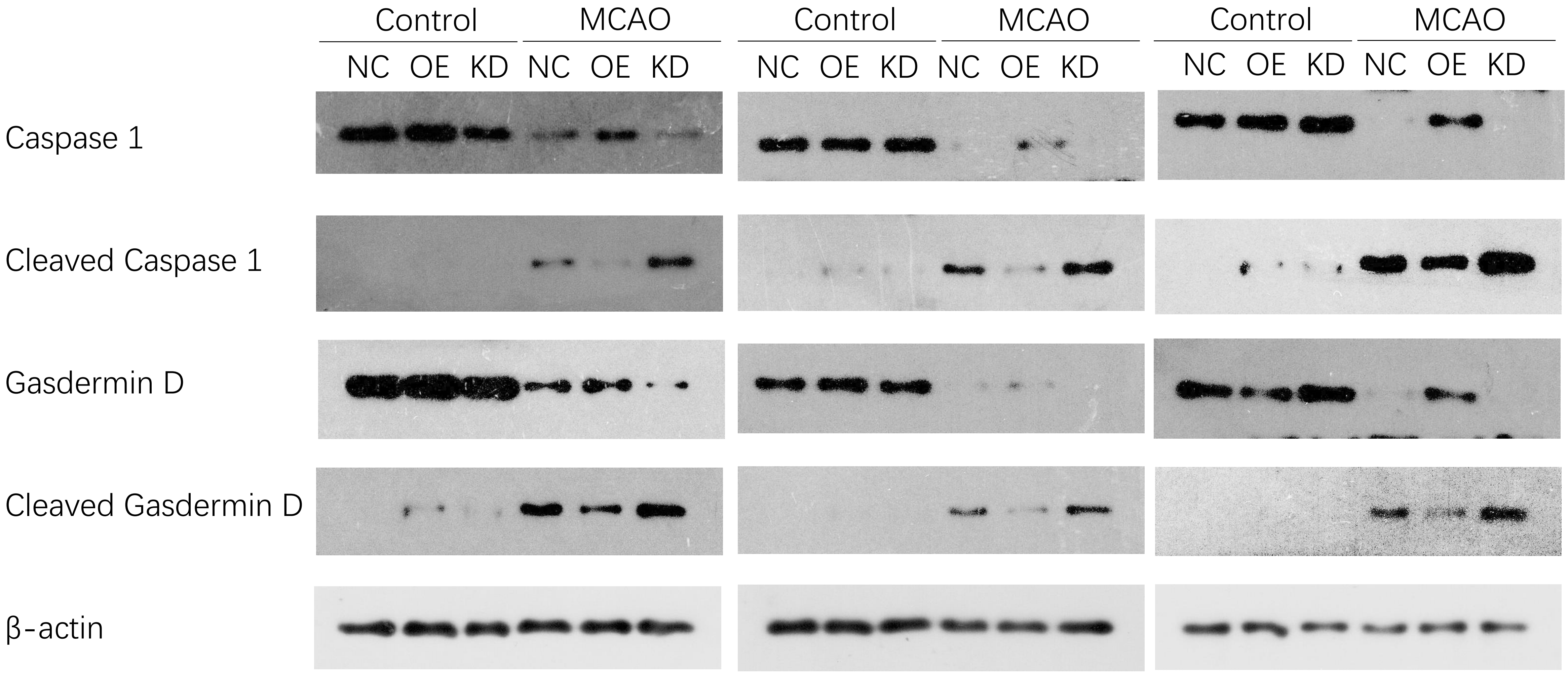

Figure 3 Supplement

Western blot—HAX-1 expression in Microglia

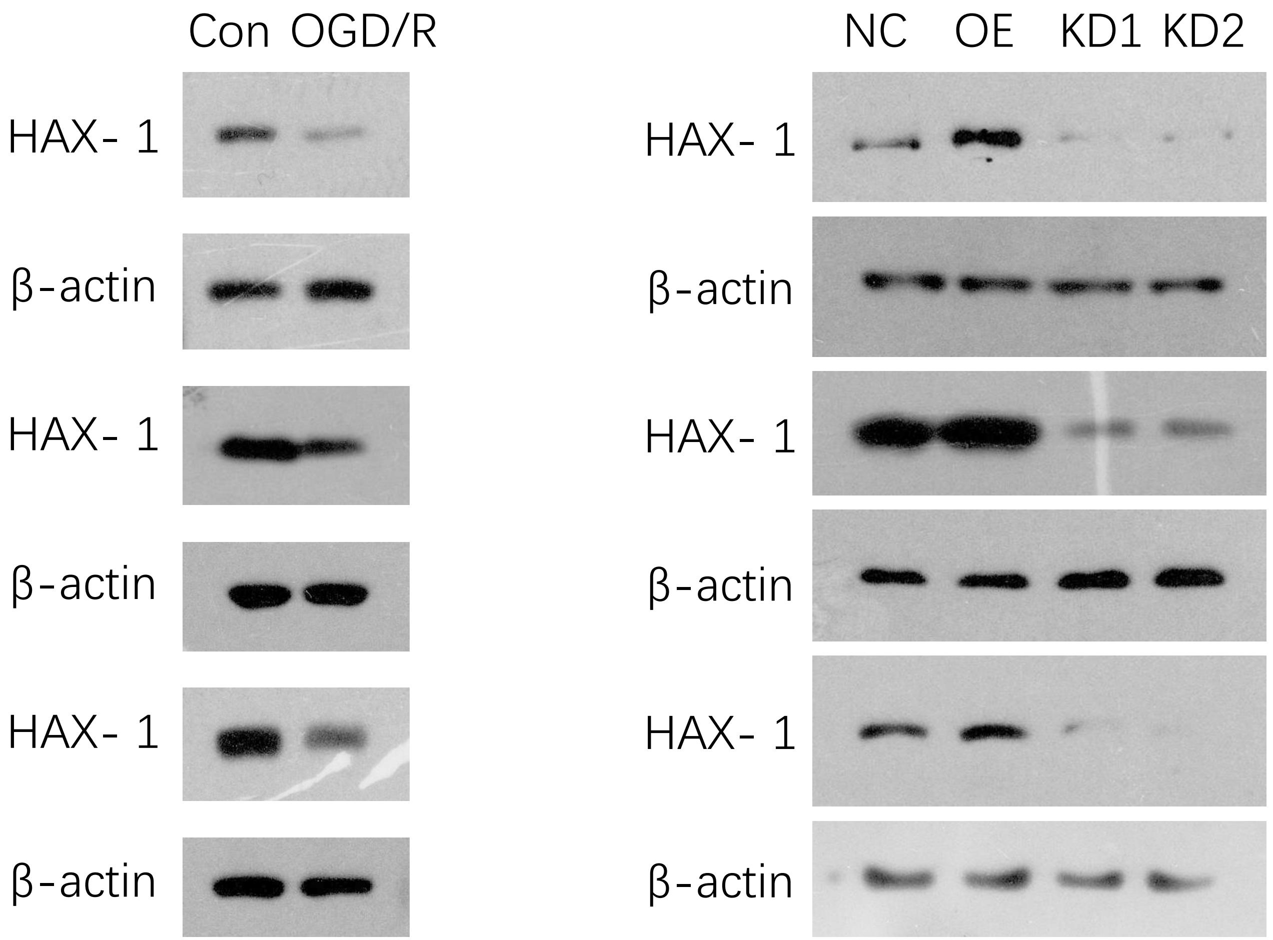

Figure 3 Supplement

Western blot—Activation of Caspase1/GasderminD in Microglia

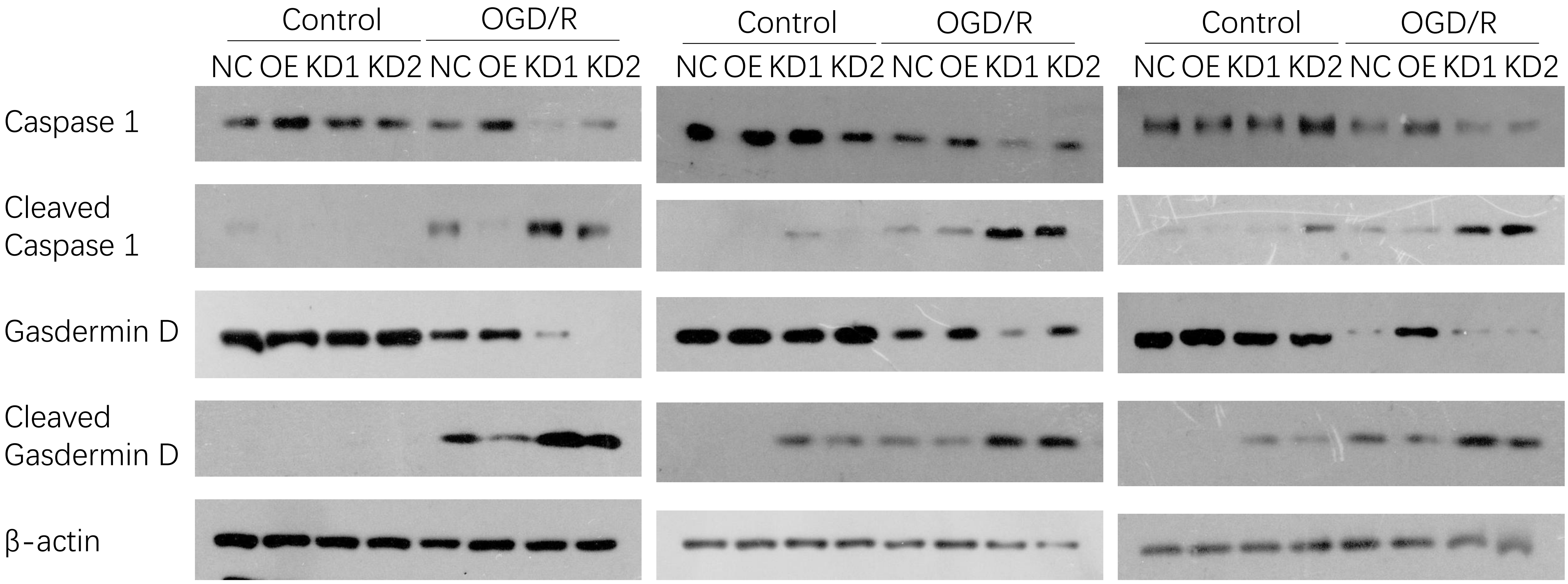

Figure 4 Supplement

Western blot—MCC950 blocked HAX-1 induced activation of Caspase1/GasderminD

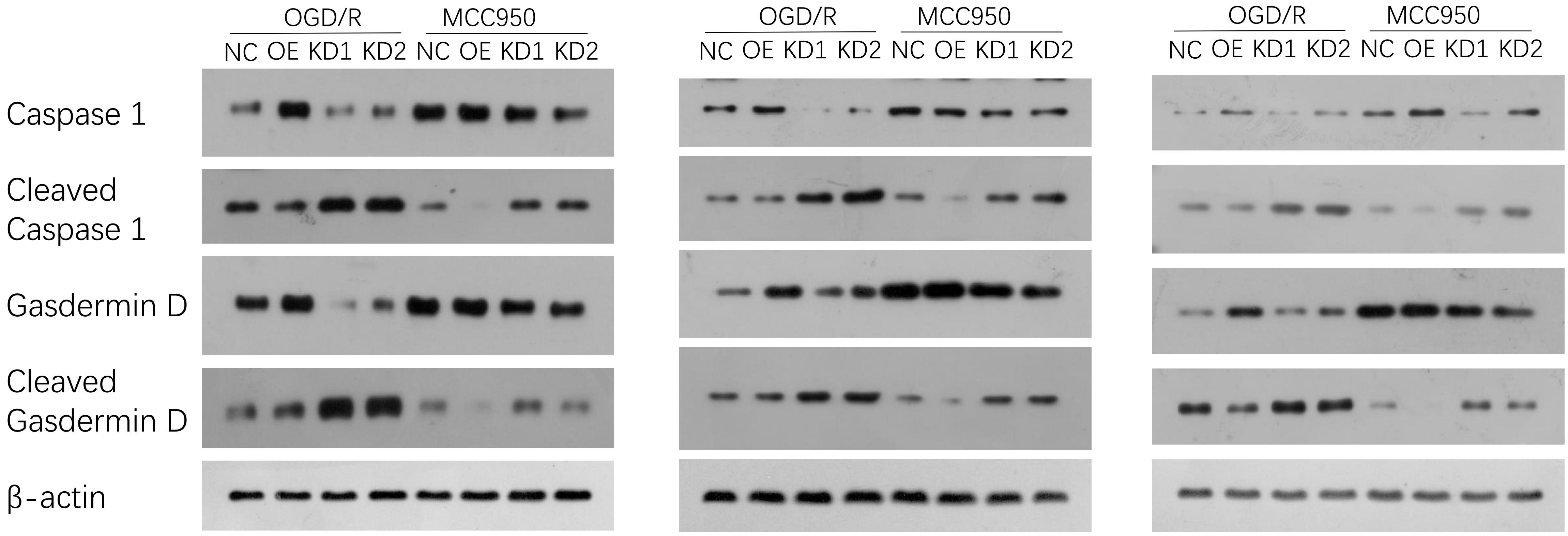

Figure 4 Supplement

Co-IP detected the combination of HAX-1 and NLRP3

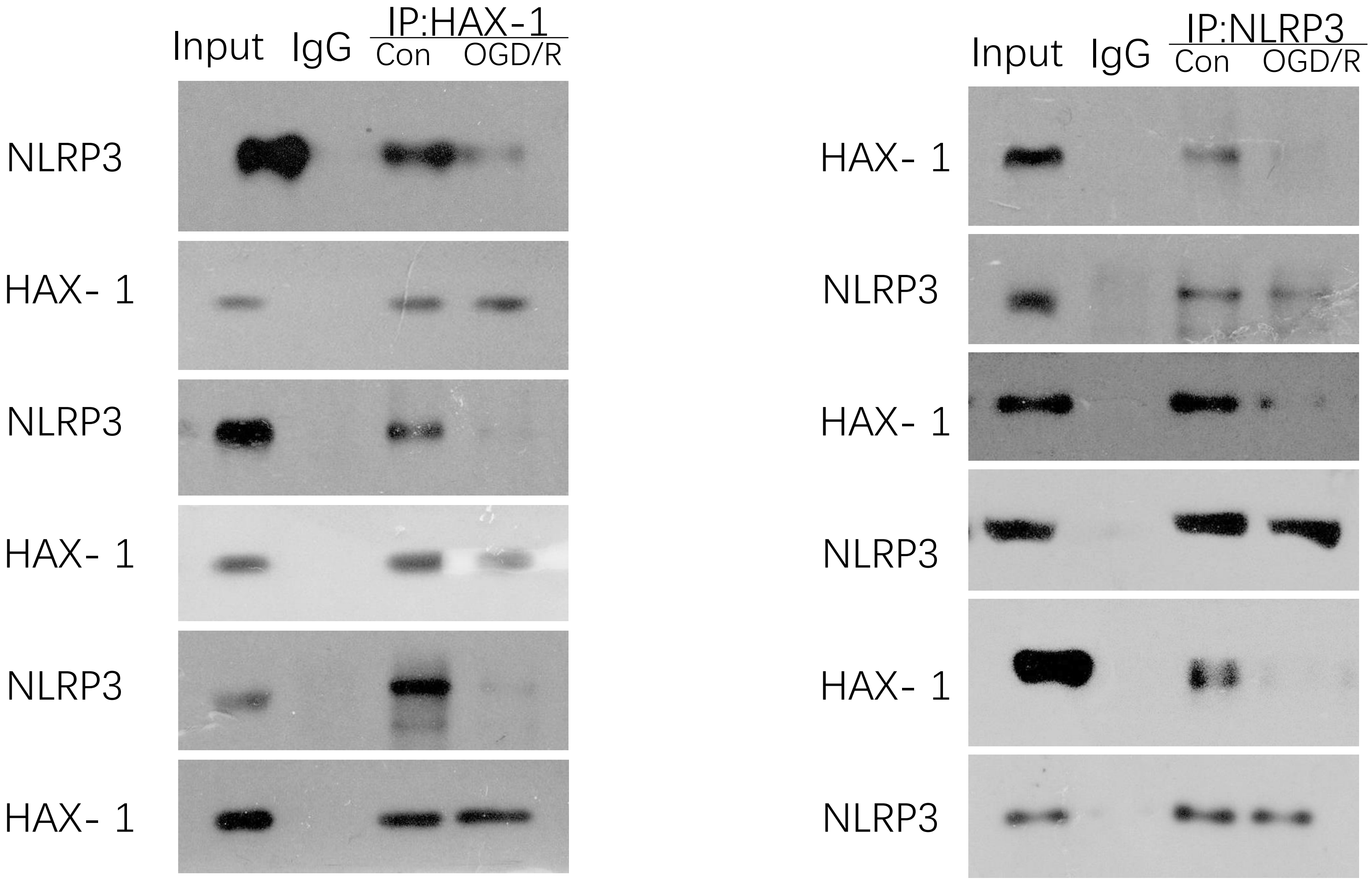

Figure 4 Supplement

Co-IP detected the interaction of NLRP3 and ASC

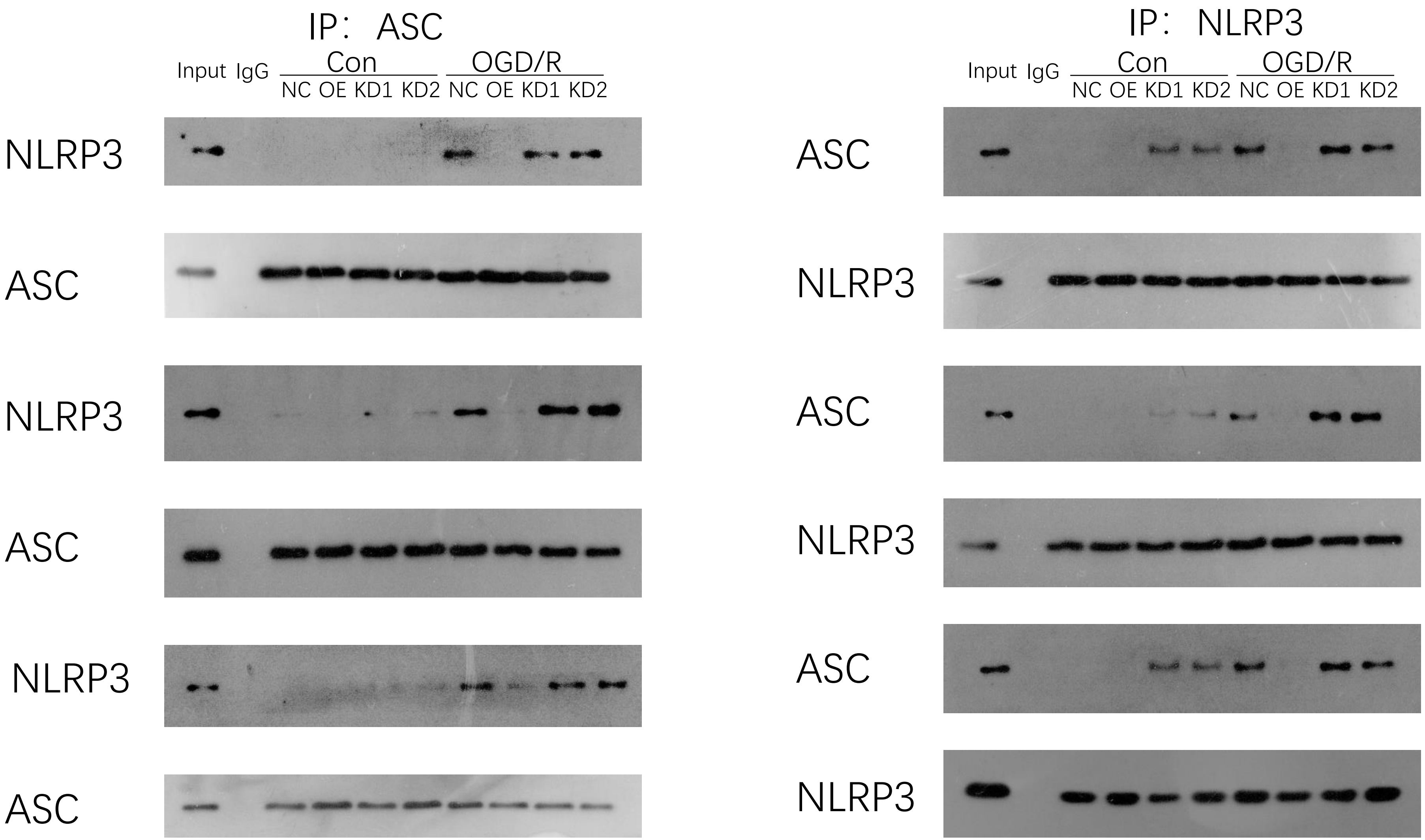

Figure 5 Supplement

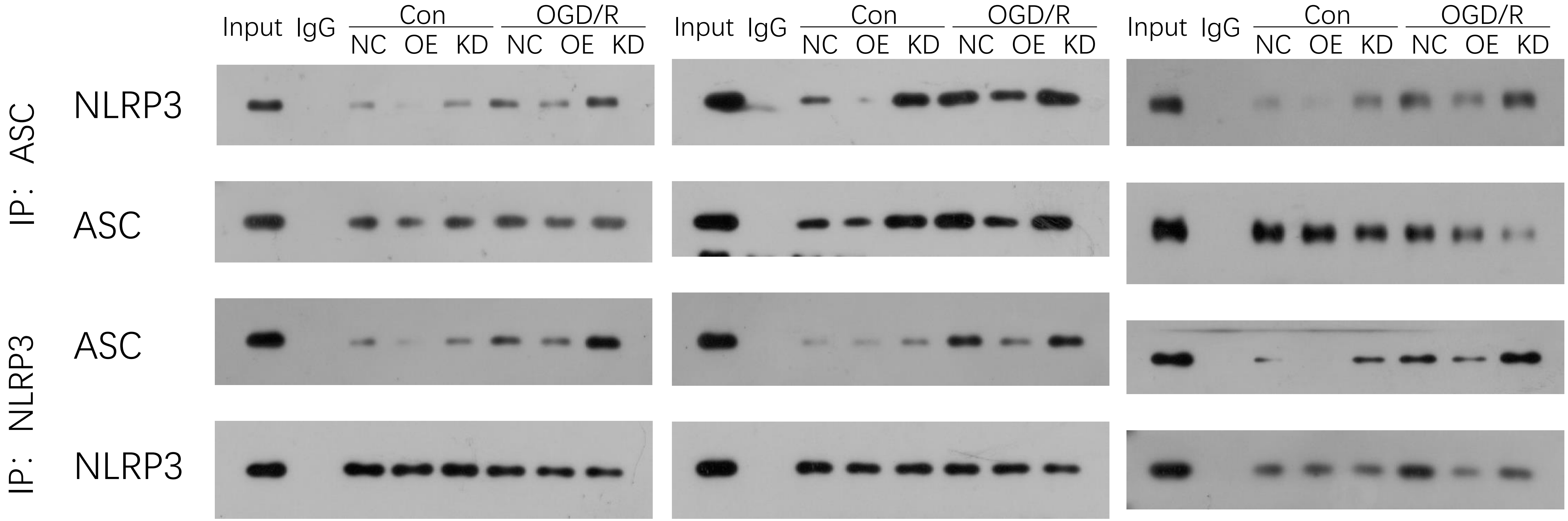

Supplement: Supplementary file 1 — Supplement materials [file 41420_2024_2005_MOESM1_ESM.pdf]
